# Two antibodies show broad, synergistic neutralization against SARS-CoV-2 variants by inducing conformational change within the RBD

**DOI:** 10.1093/procel/pwad040

**Published:** 2023-07-20

**Authors:** Hui Sun, Tingting Deng, Yali Zhang, Yanling Lin, Yanan Jiang, Yichao Jiang, Yang Huang, Shuo Song, Lingyan Cui, Tingting Li, Hualong Xiong, Miaolin Lan, Liqin Liu, Yu Li, Qianjiao Fang, Kunyu Yu, Wenling Jiang, Lizhi Zhou, Yuqiong Que, Tianying Zhang, Quan Yuan, Tong Cheng, Zheng Zhang, Hai Yu, Jun Zhang, Wenxin Luo, Shaowei Li, Qingbing Zheng, Ying Gu, Ningshao Xia

**Affiliations:** State Key Laboratory of Molecular Vaccinology and Molecular Diagnostics, School of Public Health, School of Life Sciences, Xiamen University, Xiamen 361102, China; National Institute of Diagnostics and Vaccine Development in Infectious Diseases, School of Public Health, School of Life Sciences, Xiamen University, Xiamen 361102, China; State Key Laboratory of Vaccines for Infectious Diseases, School of Public Health, School of Life Sciences, Xiamen University, Xiamen 361102, China; State Key Laboratory of Molecular Vaccinology and Molecular Diagnostics, School of Public Health, School of Life Sciences, Xiamen University, Xiamen 361102, China; National Institute of Diagnostics and Vaccine Development in Infectious Diseases, School of Public Health, School of Life Sciences, Xiamen University, Xiamen 361102, China; State Key Laboratory of Vaccines for Infectious Diseases, School of Public Health, School of Life Sciences, Xiamen University, Xiamen 361102, China; State Key Laboratory of Molecular Vaccinology and Molecular Diagnostics, School of Public Health, School of Life Sciences, Xiamen University, Xiamen 361102, China; National Institute of Diagnostics and Vaccine Development in Infectious Diseases, School of Public Health, School of Life Sciences, Xiamen University, Xiamen 361102, China; State Key Laboratory of Vaccines for Infectious Diseases, School of Public Health, School of Life Sciences, Xiamen University, Xiamen 361102, China; Xiang An Biomedicine Laboratory, Xiamen 361102, China; State Key Laboratory of Molecular Vaccinology and Molecular Diagnostics, School of Public Health, School of Life Sciences, Xiamen University, Xiamen 361102, China; National Institute of Diagnostics and Vaccine Development in Infectious Diseases, School of Public Health, School of Life Sciences, Xiamen University, Xiamen 361102, China; State Key Laboratory of Vaccines for Infectious Diseases, School of Public Health, School of Life Sciences, Xiamen University, Xiamen 361102, China; State Key Laboratory of Molecular Vaccinology and Molecular Diagnostics, School of Public Health, School of Life Sciences, Xiamen University, Xiamen 361102, China; National Institute of Diagnostics and Vaccine Development in Infectious Diseases, School of Public Health, School of Life Sciences, Xiamen University, Xiamen 361102, China; State Key Laboratory of Vaccines for Infectious Diseases, School of Public Health, School of Life Sciences, Xiamen University, Xiamen 361102, China; State Key Laboratory of Molecular Vaccinology and Molecular Diagnostics, School of Public Health, School of Life Sciences, Xiamen University, Xiamen 361102, China; National Institute of Diagnostics and Vaccine Development in Infectious Diseases, School of Public Health, School of Life Sciences, Xiamen University, Xiamen 361102, China; State Key Laboratory of Vaccines for Infectious Diseases, School of Public Health, School of Life Sciences, Xiamen University, Xiamen 361102, China; State Key Laboratory of Molecular Vaccinology and Molecular Diagnostics, School of Public Health, School of Life Sciences, Xiamen University, Xiamen 361102, China; National Institute of Diagnostics and Vaccine Development in Infectious Diseases, School of Public Health, School of Life Sciences, Xiamen University, Xiamen 361102, China; State Key Laboratory of Vaccines for Infectious Diseases, School of Public Health, School of Life Sciences, Xiamen University, Xiamen 361102, China; Institute for Hepatology, National Clinical Research Center for Infectious Disease, Shenzhen Third People’s Hospital, School of Medicine, Southern University of Science and Technology, Shenzhen 518112, China; The Second Affiliated Hospital, School of Medicine, Southern University of Science and Technology, Shenzhen 518112, China; State Key Laboratory of Molecular Vaccinology and Molecular Diagnostics, School of Public Health, School of Life Sciences, Xiamen University, Xiamen 361102, China; National Institute of Diagnostics and Vaccine Development in Infectious Diseases, School of Public Health, School of Life Sciences, Xiamen University, Xiamen 361102, China; State Key Laboratory of Vaccines for Infectious Diseases, School of Public Health, School of Life Sciences, Xiamen University, Xiamen 361102, China; State Key Laboratory of Molecular Vaccinology and Molecular Diagnostics, School of Public Health, School of Life Sciences, Xiamen University, Xiamen 361102, China; National Institute of Diagnostics and Vaccine Development in Infectious Diseases, School of Public Health, School of Life Sciences, Xiamen University, Xiamen 361102, China; State Key Laboratory of Vaccines for Infectious Diseases, School of Public Health, School of Life Sciences, Xiamen University, Xiamen 361102, China; Xiang An Biomedicine Laboratory, Xiamen 361102, China; State Key Laboratory of Molecular Vaccinology and Molecular Diagnostics, School of Public Health, School of Life Sciences, Xiamen University, Xiamen 361102, China; National Institute of Diagnostics and Vaccine Development in Infectious Diseases, School of Public Health, School of Life Sciences, Xiamen University, Xiamen 361102, China; State Key Laboratory of Vaccines for Infectious Diseases, School of Public Health, School of Life Sciences, Xiamen University, Xiamen 361102, China; Xiang An Biomedicine Laboratory, Xiamen 361102, China; State Key Laboratory of Molecular Vaccinology and Molecular Diagnostics, School of Public Health, School of Life Sciences, Xiamen University, Xiamen 361102, China; National Institute of Diagnostics and Vaccine Development in Infectious Diseases, School of Public Health, School of Life Sciences, Xiamen University, Xiamen 361102, China; State Key Laboratory of Vaccines for Infectious Diseases, School of Public Health, School of Life Sciences, Xiamen University, Xiamen 361102, China; State Key Laboratory of Molecular Vaccinology and Molecular Diagnostics, School of Public Health, School of Life Sciences, Xiamen University, Xiamen 361102, China; National Institute of Diagnostics and Vaccine Development in Infectious Diseases, School of Public Health, School of Life Sciences, Xiamen University, Xiamen 361102, China; State Key Laboratory of Vaccines for Infectious Diseases, School of Public Health, School of Life Sciences, Xiamen University, Xiamen 361102, China; State Key Laboratory of Molecular Vaccinology and Molecular Diagnostics, School of Public Health, School of Life Sciences, Xiamen University, Xiamen 361102, China; National Institute of Diagnostics and Vaccine Development in Infectious Diseases, School of Public Health, School of Life Sciences, Xiamen University, Xiamen 361102, China; State Key Laboratory of Vaccines for Infectious Diseases, School of Public Health, School of Life Sciences, Xiamen University, Xiamen 361102, China; State Key Laboratory of Molecular Vaccinology and Molecular Diagnostics, School of Public Health, School of Life Sciences, Xiamen University, Xiamen 361102, China; National Institute of Diagnostics and Vaccine Development in Infectious Diseases, School of Public Health, School of Life Sciences, Xiamen University, Xiamen 361102, China; State Key Laboratory of Vaccines for Infectious Diseases, School of Public Health, School of Life Sciences, Xiamen University, Xiamen 361102, China; State Key Laboratory of Molecular Vaccinology and Molecular Diagnostics, School of Public Health, School of Life Sciences, Xiamen University, Xiamen 361102, China; National Institute of Diagnostics and Vaccine Development in Infectious Diseases, School of Public Health, School of Life Sciences, Xiamen University, Xiamen 361102, China; State Key Laboratory of Vaccines for Infectious Diseases, School of Public Health, School of Life Sciences, Xiamen University, Xiamen 361102, China; State Key Laboratory of Molecular Vaccinology and Molecular Diagnostics, School of Public Health, School of Life Sciences, Xiamen University, Xiamen 361102, China; National Institute of Diagnostics and Vaccine Development in Infectious Diseases, School of Public Health, School of Life Sciences, Xiamen University, Xiamen 361102, China; State Key Laboratory of Vaccines for Infectious Diseases, School of Public Health, School of Life Sciences, Xiamen University, Xiamen 361102, China; State Key Laboratory of Molecular Vaccinology and Molecular Diagnostics, School of Public Health, School of Life Sciences, Xiamen University, Xiamen 361102, China; National Institute of Diagnostics and Vaccine Development in Infectious Diseases, School of Public Health, School of Life Sciences, Xiamen University, Xiamen 361102, China; State Key Laboratory of Vaccines for Infectious Diseases, School of Public Health, School of Life Sciences, Xiamen University, Xiamen 361102, China; Xiang An Biomedicine Laboratory, Xiamen 361102, China; State Key Laboratory of Molecular Vaccinology and Molecular Diagnostics, School of Public Health, School of Life Sciences, Xiamen University, Xiamen 361102, China; National Institute of Diagnostics and Vaccine Development in Infectious Diseases, School of Public Health, School of Life Sciences, Xiamen University, Xiamen 361102, China; State Key Laboratory of Vaccines for Infectious Diseases, School of Public Health, School of Life Sciences, Xiamen University, Xiamen 361102, China; Xiang An Biomedicine Laboratory, Xiamen 361102, China; State Key Laboratory of Molecular Vaccinology and Molecular Diagnostics, School of Public Health, School of Life Sciences, Xiamen University, Xiamen 361102, China; National Institute of Diagnostics and Vaccine Development in Infectious Diseases, School of Public Health, School of Life Sciences, Xiamen University, Xiamen 361102, China; State Key Laboratory of Vaccines for Infectious Diseases, School of Public Health, School of Life Sciences, Xiamen University, Xiamen 361102, China; Xiang An Biomedicine Laboratory, Xiamen 361102, China; State Key Laboratory of Molecular Vaccinology and Molecular Diagnostics, School of Public Health, School of Life Sciences, Xiamen University, Xiamen 361102, China; National Institute of Diagnostics and Vaccine Development in Infectious Diseases, School of Public Health, School of Life Sciences, Xiamen University, Xiamen 361102, China; State Key Laboratory of Vaccines for Infectious Diseases, School of Public Health, School of Life Sciences, Xiamen University, Xiamen 361102, China; Xiang An Biomedicine Laboratory, Xiamen 361102, China; State Key Laboratory of Molecular Vaccinology and Molecular Diagnostics, School of Public Health, School of Life Sciences, Xiamen University, Xiamen 361102, China; National Institute of Diagnostics and Vaccine Development in Infectious Diseases, School of Public Health, School of Life Sciences, Xiamen University, Xiamen 361102, China; State Key Laboratory of Vaccines for Infectious Diseases, School of Public Health, School of Life Sciences, Xiamen University, Xiamen 361102, China; Xiang An Biomedicine Laboratory, Xiamen 361102, China; Institute for Hepatology, National Clinical Research Center for Infectious Disease, Shenzhen Third People’s Hospital, School of Medicine, Southern University of Science and Technology, Shenzhen 518112, China; The Second Affiliated Hospital, School of Medicine, Southern University of Science and Technology, Shenzhen 518112, China; State Key Laboratory of Molecular Vaccinology and Molecular Diagnostics, School of Public Health, School of Life Sciences, Xiamen University, Xiamen 361102, China; National Institute of Diagnostics and Vaccine Development in Infectious Diseases, School of Public Health, School of Life Sciences, Xiamen University, Xiamen 361102, China; State Key Laboratory of Vaccines for Infectious Diseases, School of Public Health, School of Life Sciences, Xiamen University, Xiamen 361102, China; Xiang An Biomedicine Laboratory, Xiamen 361102, China; State Key Laboratory of Molecular Vaccinology and Molecular Diagnostics, School of Public Health, School of Life Sciences, Xiamen University, Xiamen 361102, China; National Institute of Diagnostics and Vaccine Development in Infectious Diseases, School of Public Health, School of Life Sciences, Xiamen University, Xiamen 361102, China; State Key Laboratory of Vaccines for Infectious Diseases, School of Public Health, School of Life Sciences, Xiamen University, Xiamen 361102, China; Xiang An Biomedicine Laboratory, Xiamen 361102, China; State Key Laboratory of Molecular Vaccinology and Molecular Diagnostics, School of Public Health, School of Life Sciences, Xiamen University, Xiamen 361102, China; National Institute of Diagnostics and Vaccine Development in Infectious Diseases, School of Public Health, School of Life Sciences, Xiamen University, Xiamen 361102, China; State Key Laboratory of Vaccines for Infectious Diseases, School of Public Health, School of Life Sciences, Xiamen University, Xiamen 361102, China; Xiang An Biomedicine Laboratory, Xiamen 361102, China; State Key Laboratory of Molecular Vaccinology and Molecular Diagnostics, School of Public Health, School of Life Sciences, Xiamen University, Xiamen 361102, China; National Institute of Diagnostics and Vaccine Development in Infectious Diseases, School of Public Health, School of Life Sciences, Xiamen University, Xiamen 361102, China; State Key Laboratory of Vaccines for Infectious Diseases, School of Public Health, School of Life Sciences, Xiamen University, Xiamen 361102, China; Xiang An Biomedicine Laboratory, Xiamen 361102, China; State Key Laboratory of Molecular Vaccinology and Molecular Diagnostics, School of Public Health, School of Life Sciences, Xiamen University, Xiamen 361102, China; National Institute of Diagnostics and Vaccine Development in Infectious Diseases, School of Public Health, School of Life Sciences, Xiamen University, Xiamen 361102, China; State Key Laboratory of Vaccines for Infectious Diseases, School of Public Health, School of Life Sciences, Xiamen University, Xiamen 361102, China; Xiang An Biomedicine Laboratory, Xiamen 361102, China; State Key Laboratory of Molecular Vaccinology and Molecular Diagnostics, School of Public Health, School of Life Sciences, Xiamen University, Xiamen 361102, China; National Institute of Diagnostics and Vaccine Development in Infectious Diseases, School of Public Health, School of Life Sciences, Xiamen University, Xiamen 361102, China; State Key Laboratory of Vaccines for Infectious Diseases, School of Public Health, School of Life Sciences, Xiamen University, Xiamen 361102, China; Xiang An Biomedicine Laboratory, Xiamen 361102, China; State Key Laboratory of Molecular Vaccinology and Molecular Diagnostics, School of Public Health, School of Life Sciences, Xiamen University, Xiamen 361102, China; National Institute of Diagnostics and Vaccine Development in Infectious Diseases, School of Public Health, School of Life Sciences, Xiamen University, Xiamen 361102, China; State Key Laboratory of Vaccines for Infectious Diseases, School of Public Health, School of Life Sciences, Xiamen University, Xiamen 361102, China; Xiang An Biomedicine Laboratory, Xiamen 361102, China; Research Unit of Frontier Technology of Structural Vaccinology, Chinese Academy of Medical Sciences, Xiamen 361102, China

**Keywords:** SARS-CoV-2, broad neutralizing antibody, rearrangement, synergistic neutralization

## Abstract

Continual evolution of the severe acute respiratory syndrome coronavirus (SARS-CoV-2) virus has allowed for its gradual evasion of neutralizing antibodies (nAbs) produced in response to natural infection or vaccination. The rapid nature of these changes has incited a need for the development of superior broad nAbs (bnAbs) and/or the rational design of an antibody cocktail that can protect against the mutated virus strain. Here, we report two angiotensin-converting enzyme 2 competing nAbs—8H12 and 3E2—with synergistic neutralization but evaded by some Omicron subvariants. Cryo-electron microscopy reveals the two nAbs synergistic neutralizing virus through a rigorous pairing permitted by rearrangement of the 472–489 loop in the receptor-binding domain to avoid steric clashing. Bispecific antibodies based on these two nAbs tremendously extend the neutralizing breadth and restore neutralization against recent variants including currently dominant XBB.1.5. Together, these findings expand our understanding of the potential strategies for the neutralization of SARS-CoV-2 variants toward the design of broad-acting antibody therapeutics and vaccines.

## Introduction

With over 760 million confirmed cases and 6.9 million deaths globally (WHO., 2023), the severe acute respiratory syndrome coronavirus 2 (SARS-CoV-2) pandemic and resultant coronavirus disease 2019 (COVID-19) continues to threaten global public health and economic recovery. Rapid viral mutagenesis has resulted in the appearance of many variants of concern (VOCs) such as Beta, Delta, Omicron, and various Omicron sublineages (Cocherie et al. 2022, Desingu and Nagarajan 2022, Qu et al., 2023b , Tegally et al. 2022, Wang et al. 2022), which currently dominated by Omicron subvariant XBB.1.5 (Qu et al, 2023a, Uraki et al. 2023, Yue et al. 2023, Zhu et al. 2023). The substantial and rapid development of mutations on the receptor-binding domain (RBD) of the viral spike protein has resulted in the diminished efficacy of currently available vaccines and immune therapies, as most were developed against the wild-type (WT) or early emergent variants (Cao et al. 2022a, b, Cele et al. 2022, Iketani et al. 2022, Tuekprakhon et al. 2022, Wang et al. 2022b). The further evolution of the virus and the resultant unpredictability of this pandemic require the continued design and development of next generation, superior, and cross-protective therapeutics and vaccines.

Neutralizing antibodies (nAbs) are effective countermeasures against the continuously evolving SARS-CoV-2 virus: not only for antibody-based immunotherapy but also for understanding and defining the conservation of epitopes that may be used in the development of vaccines less likely to be hampered by frequent spike protein mutations (Crowe 2022). Yet, most previously approved antibody therapeutics, such as REGEN-COV2 (Regeneron), Bamlanivimab/Etesevimab (Lilly), and Bebtelovimab (LY-CoV1404, Lilly), have abolished efficacies against current variants without exception (Cao et al. 2022b, Cavazzoni 2022, Iketani et al. 2022, Miller et al. 2023, Planas et al. 2023). Although with diminished neutralization against newly emerging variants for most available nAbs, the combination of those nAbs by way of bispecific construction, has been demonstrated to extend the neutralizing breadth to some extent (Ku et al. 2022, Li et al. 2022, Miersch et al. 2022, Wang et al. 2022c). This information together indicates that the rational design of antibodies and/or their cocktails toward broad neutralization is still desired.

In this study, we generated two SARS-CoV-2 nAbs with potent and synergetic binding and neutralization efficiencies against various SARS-CoV-2 variants including early Omicron sublineages. Cryo-electron microscopy (cryo-EM) structures of the mono- and double-antibody-bound immune complexes reveal an antibody-binding-induced rearrangement of the RBD. Such rearrangement of RBD is further demonstrated a common phenomenon, which inspire us to construct the bispecific antibodies based on the two nAbs. The resultant bispecific nAbs tremendously extend the neutralizing breadth and restore neutralization against XBB variants. These results expand our understanding of the dynamics of spike protein and the broad neutralization of nAbs, which will guide the rational design of antibody therapeutics and vaccines for combating the continually evolving virus.

## Results

### Synergetic neutralization of two angiotensin-converting enzyme 2-competing antibodies

**Figure 1.**
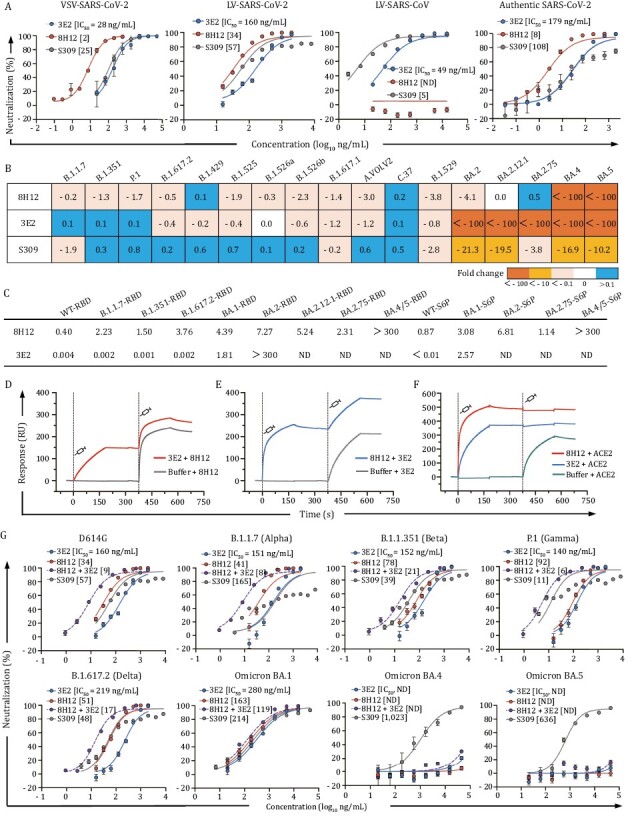
**Characterization of SARS-CoV-2 nAbs 8H12 and 3E12**. (A) Neutralizing activities of 8H12 and 3E2 against pseudotyped SARS-CoV-2 and SARS-CoV viruses as well as authentic SARS-CoV-2 virus. S309 is used as a positive control. (B) Fold change in the neutralization efficacy of the two nAbs and S309 control against SARS-CoV-2 variants as compared with the D614G stain. (C) Binding affinities of 8H12 and 3E2 to diverse spike proteins and RBDs. *K*_D_ values (nmol/L) are shown. (D and E) Simultaneous binding potentials of 8H12 and 3E2 are tested using SPR. (F) SPR analysis confirms the ACE2 blocking potential of both 8H12 and 3E2. (G) Synergistic neutralizing efficacies of double-antibody combination against the pseudotyped SARS-CoV-2 D614G strain and variants of concern (VOCs). ND means not detectable.

The two SARS-CoV-2 monoclonal antibodies (mAbs), 8H12 and 3E2, were generated from mice immunized with recombinant spike proteins (S2P) of SARS-CoV-2 and SARS-CoV sequentially. The neutralizing activities of two mAbs were evaluated using vesicular stomatitis virus (VSV)-based prototype SARS-CoV-2 pseudovirus (VSV-SARS-CoV-2) and lentivirus (LV)-based prototype SARS-CoV-2 (LV-SARS-CoV-2) and SARS-CoV (LV-SARS-CoV) pseudoviruses, as well as the authentic SARS-CoV-2 virus (Fig. 1A). Both mAbs exhibited potent neutralization efficacies against the prototyped SARS-CoV-2 with median inhibitory concentrations (IC_50_) of 34–160 ng/mL, with 3E2 further conferring cross-neutralizing activity against SARS-CoV (IC_50_: 49 ng/mL) (Fig. 1A).

We next assessed the neutralizing breadths of nAbs against LV-based pseudoviruses of SARS-CoV-2 variants, including previous VOCs/VOIs and the current circulating Omicron subvariants. 8H12 and 3E2 showed diverse neutralizing breadths against the variants. 8H12 could neutralize all variants that emerged before BA.4/5, whereas 3E2 was evaded by Omicron subvariants BA.2, BA2.12.1, BA.2.75, and BA.4/5 (Fig. 1B and Table S1). Despite some decreased or abolished neutralization activities, the surface plasmon resonance (SPR) validated the high binding affinity of 8H12 against all tested spike proteins of the Omicron subvariants, except for the BA.4/5 spike (Figs. 1C and S1). Of note, although showed abolished neutralization against BA.4/5, 8H12 remain showed decreased but not eliminated affinity to BA.4/5 (Figs. 1C and S1). In contrast, 3E2 showed excellent binding affinities only to variants before BA.2 (Figs. 1C and S1). Binding assays further confirmed the diverse binding efficacies of two nAbs to the different spike proteins. (Figs. 1C and S2).

We noted in the SPR analysis that the combinations of two nAbs could simultaneously bind to the spike protein (Fig. 1D and 1E), which suggested that the two nAbs recognized diverse epitopes through noncompetitive binding to the RBD. Additionally, 8H12 and 3E2 each could completely block the binding of angiotensin-converting enzyme 2 (ACE2) to the RBD (Fig. 1F). It is an established fact that the combination of two or more antibodies targeting distinct epitopes can enhance therapeutic efficacy and resist viral escape (Copin et al. 2021, Wang et al. 2022d, Xiong et al. 2022, Zhang et al. 2021). Thus, we next sought to evaluate the synergetic neutralization of two nAbs by the LV-based pseudoviruses. The double-antibody cocktail indeed displayed a significant synergistic effect against D614G strain with IC_50_ of 9 ng/mL, an improvement of ~4- to 18-fold as compared with that of the single antibody (Fig. 1G). The double-antibody cocktail also showed similar synergistic neutralizing efficacies to the Alpha, Beta, Gamma, Delta, and original Omicron variants, but not to the BA.4/5 variant, probably due to the diminished neutralization activity of nAbs 8H12 and 3E2 to this variant (Table S1). These data demonstrated the synergetic neutralization potential of double-antibody cocktails against SARS-CoV-2 and most of its variants.

### Cryo-EM structures of immune complexes

**Figure 2.**
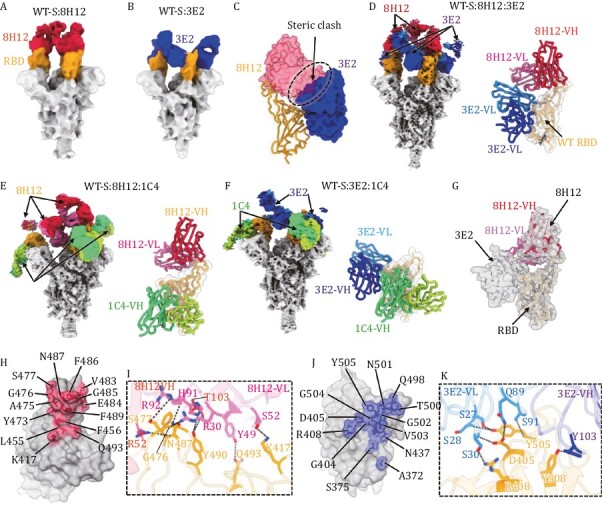
**Cryo-EM analysis of single- and double-antibody-bound spike immune complexes**. (A and B) Cryo-EM density maps of the wild-type spike protein (WT-S) bound with 8H12 (A) and 3E2 (B). The RBDs are colored in orange and the nAbs 8H12 and 3E2 are colored in red and blue, respectively. (C) Potent steric clashing between 8H12 and 3E2 when binding together are indicated as black dashes. Established models of the RBD (shown as ribbon) and the variable domains of the two nAbs (shown as surface) are shown based on the structures of the mono-antibody immune complexes. (D–F) Structures of the double-antibody-bound spike immune complexes. Cryo-EM density maps (left) and models of the antibody interface (right) of immune complexes of WT-S:8H12:3E2 (D), WT-S:8H12:1C4 (E), and WT-S:3E2:1C4 (F) are shown with the RBD and Fabs colored by domain. (G) Simultaneous binding of 8H12 and 3E2 induces binding orientation change of 8H12. Localized reconstructed map of WT-S:8H12:3E2 are shown as transparent gray surface, the model of RBD:8H12 reconstructed from the WT-S:8H12:1C4 is fitted and show different binding orientations of 8H12. (H–K) The footprints and interaction details of 8H12 (H and I) and 3E2 (J and K). The footprints of the nAbs on the RBD are colored accordingly and shown in transparent surface representation, with the epitope residues labeled and shown in stick representation. Residues involved in hydrogen bonding interactions (indicated as black dashes) are labeled and highlighted.

To investigate the molecular basis for the synergetic neutralization of these two nAbs, we first determined the mono-antibody-bound WT spike (WT-S) immune complexes. Two medium-resolution cryo-EM structures revealed distinct binding modes of each nAbs, with structural superimposition indicating potential steric hindrance between nAbs 8H12 and 3E2 (Figs. 2A–C, S3 and Table S2); albeit the SPR results showed no such competition of binding (Fig. 1D and 1E). Thus, we next validated the simultaneous binding of double-antibody combination by cryo-EM analysis, with structure of WT-S:8H12:3E2 obtained at resolutions of 3.58 Å (Figs. 2D and S4). In order to elucidate the potent conformational changes induced by the simultaneous binding of 8H12 and 3E2, we next sought to improve the reconstruction resolution of WT-S:8H12 and WT-S:3E2. To this end, a third antibody 1C4 was included to cryo-EM reconstruction of 8H12- and 3E2-based double-antibody immune complexes and finally achieve to resolutions of 3.60 Å (WT-S:8H12:1C4) and 3.80 Å (WT-S:3E2:1C4), respectively (Figs. 2E, 2F and S4). Localized refinement of the RBD and variable domains of the fragment of antigen bindings (Fabs) were also performed to improve local resolutions and obtained at a resolutions of 3.59, 3.77, and 3.92 Å, respectively (Figs. 2D–F, S4 and Table S2). These structures indeed revealed the simultaneous binding potential of 8H12 and 3E2 (Fig. 2D), which cause the changing of binding orientation of 8H12 to avoid potent steric clashes between two antibodies (Fig. 2G). We further validated this phenomenon in the structures of immune complexes of BA.1-S:8H12:3E2 and BA.2-S:8H12:3E2, respectively (Figs. S5 and S6).

Generally, RBD-specific nAbs can be categorized into five classes (I–V) according to their binding modes and receptor-blocking capacity (Barnes et al. 2020). Accordingly, 8H12 and 3E2 belong to Class I and Class IV, respectively (Fig. S7). The binding mode of 8H12 resembles previously reported nAbs S2E12 (Tortorici et al. 2020), AZD-8895 (Dong et al. 2021), 58G6 (Li et al. 2021a), XMA01 (Wang et al. 2022d), and WRAIR-2125 (Dussupt et al. 2021). This type of nAb binds to the left shoulder of the RBD (Fig. S7B). The epitope of 8H12 is composed of 14 RBD residues (K417, L455, F456, Y473, A475, G476, S477, V483, E484, G485, F486, N487, F489, and Q493) mainly focused on the 472–489 loop (Fig. 2H), forming a network of eight hydrogen bonds (Fig. 2I).

As for 3E2, it mainly binds to an epitope on the opposite side of the 1C4 epitope; a similar binding mode is shown for nAbs S2X35 (Starr et al. 2021), S2X259 (Tortorici et al. 2021), VacW-209 (Ju et al. 2022), COVA1-16 (Liu et al. 2020), and CR3022 (Wu et al. 2020). The binding epitope comprises a total of 13 residues (A372, S375, G404, D405, R408, N437, Q498, T500, N501, G502, V503, G504, Y505, and Y508), of which D405, R408, Y505, and Y508 form seven hydrogen bonds mainly with the light chain of 3E2 (Fig. 2J and 2K). Superimposition of two nAbs with the ACE2 receptor shows that both 8H12 and 3E2 could sterically hinder the binding of ACE2 (Fig. S8), consistent with the result confirmed by SPR assay (Fig. 2G).

### Pairing-binding induces conformational change at one loop in the RBD

**Figure 3.**
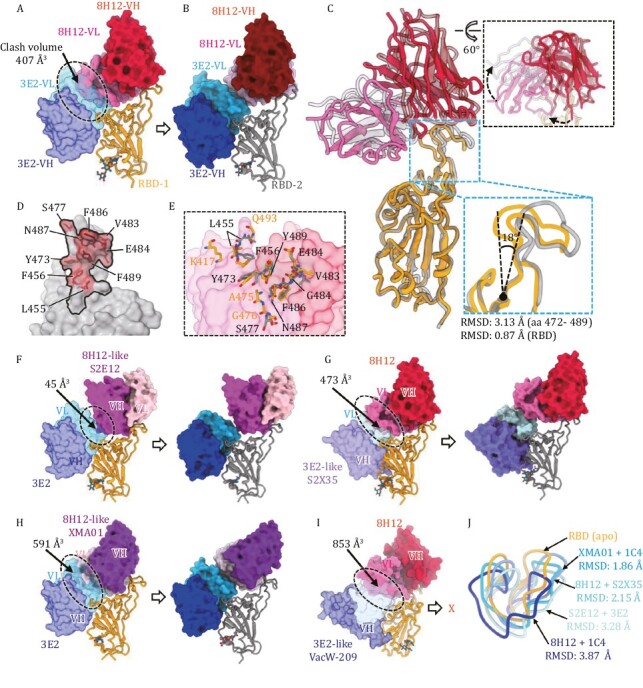
**Different antibody combinations induce RBD rearrangement**. (A) Superimposition of the binding models of RBD:3E2 (from WT-S:1C4:3E2 reconstruction) and RBD:8H12 (from WT-S:8H12:1C4 reconstruction) shows steric clash (black dashes) potency between 8H12 and 3E2. The calculated clash volume is about 407 Å^3^. (B) Model of RBD:8H12:3E2 from the WT-S:8H12:3E2 reconstruction shows no steric clashing between bound 8H12 and 3E2. (C) Superimposition of models of RBD:8H12 (from WT-S:8H12:1C4 reconstruction) and RBD:8H12 (from WT-S:8H12:3E2 reconstruction) shows structural rearrangement of the RBD and altered binding orientation of 8H12 in the structure of WT-S:8H12:1C4. (D) Comparison of the 8H12 footprints on the *apo-*RBD (black line) and on the rearranged RBD (red transparent surface representation with epitope residues showed as stick). (E) Structural details of the conformational changes of the 8H12 epitope. Models of *apo-*RBD:8H12 and rearranged RBD:8H12 are superimposed based on 8H12 (shown as transparent surface representation). The 8H12 epitope residues on the apo- and rearranged-RBDs are labeled and shown as sticks. (F) Cryo-EM analysis of WT-S in complex with 3E2 and 8H12-like S2E12. Superimposition (left) shows steric clashing between two nAbs with a calculated volume of ~45 Å^3^. In contrast, reconstruction of WT-S:3E2:S2E12 shows no clashing between the two nAbs due to rearrangement of the RBD. (G) Cryo-EM analysis of WT-S in complex with 3E2-like S2X35 and 8H12. Superimposition (left) shows steric clashing between the two nAbs, with a calculated volume of ~474 Å^3^. In contrast, reconstruction of WT-S:S2X35:8H12 shows no clashing between the two nAbs due to rearrangement of the RBD. (H) Cryo-EM analysis of WT-S in complex with 3E2 and 8H12-like XMA01. Superimposition (left) shows steric clashing between the two nAbs, with a calculated volume of ~591 Å^3^. In contrast, reconstruction of WT-S:3E2:XMA01 shows no clashing between two nAbs due to rearrangement of the RBD. (I) Cryo-EM analysis of WT-S in complex with 3E2-like VacW-209 and 8H12. Superimposition (left) shows steric clashing between the two nAbs with the largest calculated volume of ~853 Å^3^. Reconstruction of WT-S:VacW-209:8H12 shows no simultaneous binding of the two nAbs to the same RBD. (J) Superimposition of the key 472–490 loop on the rearranged-RBDs induced by different double-antibody combinations and the *apo-*RBD. The corresponding RMSDs are indicated.

We next sought to investigate the structural basis for the simultaneous binding of the two theoretically colliding nAbs, 8H12 and 3E2. In the WT-S:8H12:1C4 and WT-S:3E2:1C4 structures, the binding of the two nAbs induced no any conformational changes to the bound RBD. However, structural alignment indicated significant steric hindrance between 8H12-VL and 3E2-VL, with a calculated clash volume of ~407 Å^3^ (the variable domain of one Fab: ~4,300 Å^3^) (Fig. 3A). Yet, superimposition of WT-S:8H12:3E2 (Fig. 3B) onto the former two structures revealed obvious conformational changes to both 8H12 and its binding epitope (Fig. 3A), suggestive of 8H12/3E2 combinational binding-induced rearrangement of the bound RBD. Indeed, the structural comparison suggests that the RBD would undergo dramatic local rearrangement mainly in the aa. 472–489 loop, with a root mean square deviation (RMSD) value of 3.13 Å, and that this change would be accompanied by an outward extension of ~18° compared with the *apo-*RBD (Fig. 3C). Additionally, whereas the 3E2 binding orientation remained unchanged (Fig. S9), we noted a clockwise rotation in 8H12 that helped to accommodate the simultaneous binding of two Fabs (Fig. 3C). Furthermore, we noted a slight difference in the 8H12 binding epitopes between the two states of the 8H12-bound RBD (Fig. 3D and 3E): the 8H12 footprint on the rearranged RBD consisted of fewer (9) epitope residues, and accounted for a reduced interface area in the rearranged RBD (850.9 Å^2^ for *apo-*RBD vs. 670.5 Å^2^ for rearranged RBD). Remarkably, K417 and Q493 in the rearranged RBD had moved away from the contact interface of 8H12 but this did not affect its activity. In both cases, the bulk side chain of F486 participates in critical interactions with 8H12 via inserting itself into a hydrophobic pocket formed on the surface of 8H12 (Fig. S10), which mimics ACE2–RBD interactions among Class I/II nAbs (Barnes et al. 2020, Du et al. 2022, Wang et al. 2022d); this may explain the evasion of 8H12 to BA.4/5, as this antibody harbors an F486V mutation on the RBD.

Considering that both the nAbs 8H12 (Class I) and 3E2 (Class IV) are ACE2 blockers and showed high efficacy in blocking viral infection, we wondered whether a common phenomenon exists for such double-antibody-binding-induced RBD rearrangement. 8H12-like nAbs S2E12 (Tortorici et al. 2020), XMA01 (Wang et al. 2022d), and 3E2-like nAbs S2X35 (Starr et al. 2021), VacW-209 (Ju et al. 2022) were selected for further cryo-EM analysis (Fig. 3F–I). The combinations of 3E2/S2E12, S2X35/8H12, 3E2/XMA01, and VacW-209/8H12 showed increased calculated clash volumes ranging from 45 to 853 Å^3^, implying widespread theoretical collision between those Class I and Class IV nAbs (Fig. 3F–H). Yet, similar to our experience, all of these antibody combinations showed simultaneous binding activity to the spike trimer through SPR analysis (Fig. S11). Cryo-EM reconstructions of the WT-S in complex with different double-antibody combinations revealed that the combinations of 3E2/S2E12, S2X35/8H12, and 3E2/XMA01 can bind the RBD (Figs. 3F–H and S12). In contrast, the 3E2-like VacW-209 and 8H12 cannot bind to the same RBD simultaneously (Fig. 3I), probably due to their excessively large clash volume (853 Å^3^). Nonetheless, 8H12 and VacW-209 were able to simultaneously bind to a trimer by targeting different RBDs (Fig. S13). Superimposition of different structures of the above double-antibody immune complexes shows the diverse degree of conformational changes that can occur at this allosteric loop (RMSD ranging from 1.86 to 3.87 Å when comparing to the apo-RBD) (Fig. 3J). It seems that the larger the theoretical clash volume of the two antibodies, the more serious the structural variation, indicating the allosteric loop as being a critical region in the coordination of simultaneous binding. This structural information not only provides evidence for the existence of multiple potential cooperative mechanisms for synergetic neutralization capacity against SARS-CoV-2 but also demonstrates the rational design of antibody cocktails using Class I and Class IV nAb combinations.

### Bispecific antibodies recovery neutralization against recent variants including XBB.1.5

**Figure 4.**
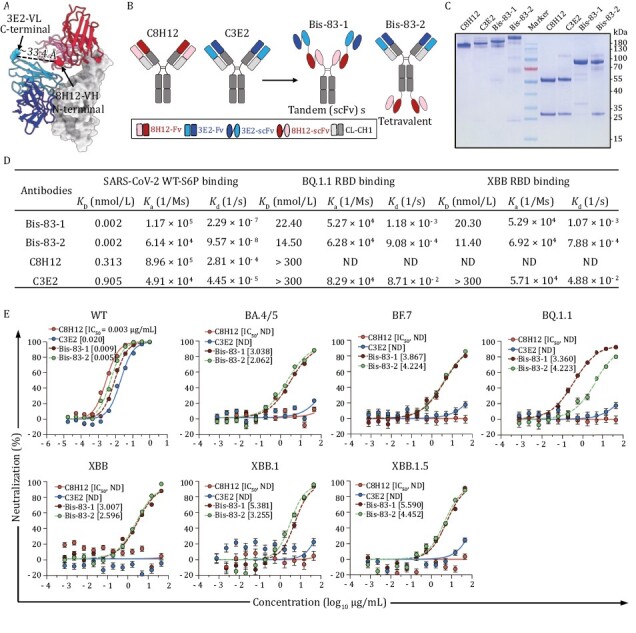
**Bispecific antibodies based on 8H12 and 3E2 show broad neutralization against Omicron subvariants.** (A) The model of RBD:8H12:3E2 shows the distance between the C-terminal of 3E2-VL and the N-terminal of 8H12-VH which is suitable for bispecific antibody engineering. (B) Schematic diagram of engineering bispecific antibodies. A tandem scFv bispecific antibody (Bis-83-1) and a tetravalent antibody (Bis-83-2) are engineered from two parental IgGs C8H12 and C3E2. (C) Bispecific antibodies identified by SDS-PAGE. Non-reduced (first four lands) and reduced SDS-PAGE (last four lands) of C8H12, C3E2, Bis-83-1, and Bis-83-2 are analyzed, respectively. the middle land is the molecular weight marker. (D) Binding affinities of bispecific antibodies bind to the RBDs of SARS-CoV-2 WT, BQ.1.1, and XBB, respectively. The parental C8H12 and C3E2 are included as comparator. (E) Neutralization potencies of the bispecific antibodies against pseudoviruses WT and Omicron subvariants. The calculated IC_50_ values are marked. ND means not detectable.

Given that the conformational changes of the RBD could be induced by the pairing binding of 8H12 and 3E2, we sought to construct the bispecific antibodies containing both 8H12 and 3E2 to enhance their efficacy as well as neutralizing breadth. Structurally, 8H12 and 3E2 are close to each other when simultaneously bind to the RBD, with a distance of ~33.4 Å between C-terminal of 3E2 VL and N-terminal of 8H12 VH (Fig. 4A), which grants the appropriate relative positions to construct a tandem scFvs format bispecific antibody that fused into human IgG1-Fc (named Bis-83-1) (Fig. 4B). Another tetravalent format bispecific antibody using the heavy chain of IgG biterminal linked with double scFvs (named Bis-83-2) was also constructed (Fig. 4B). The human IgG1-Fc chimeric formats of two mouse origin nAbs were also constructed as control and name as C8H12 and C3E2, respectively (Fig. 4B). SDS-PAGE and HPLC results confirmed the correct construction of two chimeric and two bispecific antibodies, and revealed the expected molecular weights (Figs. 4C and S14).

To test the binding properties of two bispecific antibodies, we analyzed the binding affinities of Bis-83-1 and Bis-83-2 against the WT-S protein by SPR. Compared with the parental mAbs, both Bis-83-1 and Bis-83-2 bound to WT-S protein with more than 100-fold increased binding potencies and reach picomolar level affinities (0.002 nmol/L) (Figs. 4D and S15), indicating the simultaneous binding of multiple scFvs in one antibody to the spike protein. As the fact that the BQ.1.1 and XBB RBDs showed drastically decreased or abolished binding ability to the parental C8H12 and C3E2 (Figs. 4D and S15), we next evaluated whether the binding potency of Bis-83-1 and Bis-83-2 to the RBDs of BQ.1.1 and XBB were increased. As expected, both Bis-83-1 and Bis-83-2 showed dramatically increased binding affinities against both the RBDs of BQ.1.1 and XBB (11.40–22.40 nmol/L) (Figs. 4D and S15).

We next verified whether two formats of bispecific antibodies could extend their neutralizing breadth. The pseudotyped WT SARS-CoV-2 as well as recent Omicron sublineages BA.4/5, BF.7, BQ.1.1, XBB, XBB.1, and XBB.1.5 were included in the neutralizing evaluation. Both Bis-83-1 and Bis-83-2 neutralized WT SARS-CoV-2 effectively with IC_50_ values of 0.009 and 0.005 µg/mL, respectively (Fig. 4E). Similar to BA.4/5, the BF.7, BQ.1.1 and XBBs sublineages harbor F486V/S/P mutation (Fig. S14), which devastatingly disrupt the interaction between the RBD and those Class I nAbs including 8H12 (Cao et al. 2022b, 2023, Wang et al. 2023). Additionally, D405N and R408S that emerged in BA.2 and thereafter variants resulted in abolishing the activity of 3E2 (Fig. 2D). Therefore, the two parental chimeric mAbs lost their neutralizing efficacies against all tested Omicron subvariants as expected (Fig. 4D). However, of note, the two bispecific antibodies exhibited broad neutralizing activities against all these tested Omicron subvariants including BA.4/5, BF.7, BQ.1.1, XBB, XBB.1, and XBB.1.5, with IC_50_ values ranging from 0.360 to 5.590 µg/mL (Fig. 4D), in accord with the increased binding affinities of bispecific antibodies to those variants (Fig. 4C). Taken together, these data suggested that 8H12/3E2-based bispecific antibodies with enhanced binding affinities could recover the neutralization against recent variants and extend their neutralizing breadth to achieve broad-spectrum neutralization.

## Discussion

The ongoing COVID-19 pandemic has been accompanied by a continuous evolution of SARS-CoV-2 variants. The Omicron VOCs are subvariants BA.1, BA.2, BA.4, and BA.5, which have demonstrated strong evasion from antibody therapeutics and vaccines (Cao et al. 2022a, b, Cele et al. 2022, Gruell et al. 2022, Iketani et al. 2022, Sheward et al. 2022, Tuekprakhon et al. 2022, Wang et al. 2022a). Furthermore, Omicron is continuously evolving and this has led to the emergence of various new subvariants including BA.2.75 and BA.4.6, as well as BJ.1, BQ.1.1, BF.7, and BA.2.10 (XBB) reported more recently (WHO 2022). The accumulated mutations on the RBD allow the virus to escape nAb neutralization, including by even the most potent bnAbs. For example, the currently approved antibody therapeutic, LY-CoV1404, which potently neutralizes all currently known VOCs, has recently been reported to be evaded by BJ.1, BR.1, and BQ.1.1, presumably due to mutations on R346 and/or N444 (Cao et al., 2023, Westendorf et al. 2022). This highlights a continuous need to identify broader nAbs that are effective against emerging variants.

We face a huge challenge in the utilization of nAbs to prevent and treat COVID-19. Many antibody screening and selection strategies have been raised for the generation of more potent or broader nAbs. One strategy is to develop a pan-sarbecovirus broad nAb. There are several nAbs with cross-neutralization against SARS-CoV-2 and other sarbecoviruses, such as S309 (Pinto et al. 2020), S2X259 (Tortorici et al. 2021), 7D6 (Li et al. 2021b), S2H97 (Starr et al. 2021), ADG-2 (Rappazzo et al. 2021), DH1047 (Martinez et al. 2022), as well as 3E2 in this study. However, most of these nAbs are evaded by Omicron and its subvariants. Indeed, our results showed that the SARS-CoV-2/SARS-CoV cross-neutralizing nAb 3E2 was unable to neutralize the Omicron BA.2 and thereafter variants, and therefore demonstrated a narrower breadth of efficacy than 8H12, although the latter is SARS-CoV-2-specific nAb. This suggests that nAbs with broad sarbecovirus neutralizing potencies are actually more sensitive to recent variants mutations. In other words, the conserved epitopes of pan-sarbecovirus bnAbs (e.g., 3E2’s) do not exhibit equivalent potency in action to those SARS-CoV-2-specific bnAbs (e.g., 8H12). Therefore, a better selection strategy for broad nAb candidates is still urgently needed.

One approach to limit the risk of antibody resistance and provide broad synergetic neutralizing capability is to use antibody combinations to target different viral conserved epitopes (Crowe 2022, Zost and Vogt 2022). Typically, antibody cocktail rational design tends to select non-competing nAbs (Crowe 2022, Carter and Rajpal 2022). However, our recent work on coxsackievirus described the benefit of a counterintuitive antibody cocktail containing three competing nAbs that target the vulnerable receptor-binding determinant (Zheng et al. 2022). We observed synergy among the competing nAbs through a cooperative mechanism in which the binding of one nAb added or potentiated the binding of another nAb with an overlapping epitope, and these multi-antibody functions cooperatively disrupted the coxsackievirus virions (Zheng et al. 2022). SARS-CoV-2 antibody cocktails that are approved or in development always are selected based on non-overlapping antibody designs. However, here again, we show that, despite with theoretical collision, Class I and Class IV nAbs could simultaneously bind to the RBD and confer not only synergetic neutralization but also rearrangement of the bound RBD.

It’s a fact that the high binding affinity of cell receptor ACE2 largely influence the antibody evasion by variants. In this study, we indeed demonstrated that 8H12 showed reduced but not diminished binding potencies to variants that have emerged since BA.2. However, the neutralization of 8H12 against relative variants is completely abolished, indicating that the influence of neutralizing efficacy is more sensitive than binding efficacy. Indeed, it seems that there is no any available nAb would maintain its broad-spectrum neutralizing activity against the evolving virus. Increasing the binding affinities of currently by antibody engineering, e.g., bispecific construction to increase binding avidity, would be an alternative way to achieve broad neutralization. In this study, we proved the try to broaden the neutralizing breadth of currently available nAbs by different bispecific constructions. Two formats of bispecific antibodies were demonstrated with dramatically increased binding affinity and restored neutralization against those escaped variants. Similar studies of bispecific antibodies with increased affinities and broadened neutralizing breadth were also reported by multiple groups (Ku et al. 2022, Li et al., 2022, Miersch et al. 2022, Wang et al. 2022c), indicating a realistic way for antibody engineering to broaden the neutralizing breadth. Taken together, these findings shed light on the rational design of antibody therapeutics against the continuously evolving SARS-CoV-2 viruses.

## Materials and methods

### Cell lines and virus

The H1299 cells expressing human ACE2 (H1299-huACE2) were described previously (Zhang et al. 2022) and were cultured in Dulbecco’s modified Eagle’s medium (DMEM) supplemented with 10% fetal bovine serum (FBS) and penicillin-streptomycin. Vero cells were cultured in DMEM supplemented with 10% FBS and penicillin-streptomycin. Expi293F™ cells (Gibco, Thermo Fisher Scientific) were maintained in FreeStyle 293 expression medium (Gibco). The pseudotyped LV and VSV-bearing spikes of SARS-CoV-2 variants were packaged and produced as previously described (Xiong et al. 2020, Zhang et al. 2022). Authentic SARS-CoV-2 viruses (Beta/Shenzhen/SZTH-003/2020, EPI_ISL_406594 at GISAID) (Li et al. 2021b) were used in this study.

### Recombinant proteins expression and purification

Spike proteins (residues 1–1,208) and/or RBDs (residues 319–541) of SARS-CoV-2 were obtained as previously reported (Li et al. 2021b, Ju et al. 2022). In brief, a gene encoding the ectodomain of a prefusion conformation-stabilized S protein (GenBank: MN908947, GenBank: MN908947 for SARS-CoV and SARS-CoV-2 S genes, respectively) with proline substitutions at 986 and 987, “GSAS” substitution at the S1/S2 furin cleavage site (residues 682–685), a C-terminal T4 fibritin trimerization motif, an HRV3C protease, and 8× His-Tag was synthesized and individually cloned into the pcDNA3.4 vector. The genes of other trimeric spike (WT-S6P, BA.1-S6P, BA.2-S6P, BA.2.75-S6P, and BA.4/5-S6P) and RBD (WT-RBD, BA.1-RBD, BA.2-RBD, BA.2.75-RBD, BA.4/5-RBD, BF.7-RBD, BQ.1.1-RBD, and XBB-RBD) proteins were also synthesized, of which the S6P version contained an additional four Pro substitutions (F817P, A892P, A899P, and A942P) compared with S2P. The other recombinant spike proteins (BA.2.12.1-S6P, BA.2.75-S6P, B.1.1.7-RBD, B.1.315-RBD, B.1.617.2-RBD, BA.1-RBD, and BA.2-RBD) were purchased from Sino Biological Inc. or Acro Biosystems Inc.

Recombinant protein expression was performed using Expi293F cells. Briefly, plasmids encoding for targeted proteins were transiently transfected into Expi293 cells using polyethylenimine (PEI) MW40,000 (Yeasen). Cell-free supernatants were obtained 7 days after transfection by centrifugation and filtration with a 0.22-µm filter. Subsequently, the proteins were purified using a Ni-Sepharose 6 Fast Flow (Cytiva) column and stored in a PBS buffer.

### Generation of mAbs and Fabs

8H12, 3E2, and 1C4 were obtained by mouse hybridomas preparation as described previously (Tuekprakhon et al. 2022). Ascites of the three nAbs were prepared by injecting hybridoma cells into the peritoneal cavities of pristine-primed BALB/c mice and were collected 9–12 days later and stored at −20°C. In addition, the variable domain genes of S309 (Pinto et al. 2020), S2E12 (Tortorici et al. 2020), S2X35 (Starr et al. 2021), XMA01 (Wang et al. 2022d), and VacW-209 (Ju et al. 2022) heavy and light chains were inserted into a pTT5 (Thermo Fisher Scientific) vector containing the constant region of the human IgG; and the recombinant antibodies were expressed in Expi293F cells through transient transfection. All murine (ascites) and recombinant mAbs were purified by affinity chromatography using MabSelect Sure resin (Cytiva) and were stored in PBS. To further prepare the Fab fragments, mAbs were digested with papain at 0.1% (*w*/*w*) in PBS containing 30 mmol/L l-cysteine and 50 mmol/L EDTA at 37°C for 12 h. Digestion was terminated with the addition of 20–30 mmol/L iodoacetamide. Fab fragments were then purified using protein A columns and dialyzed against PBS.

### Engineering and production of bispecific antibodies

For chimeric antibody construction, the heavy chains and light chains of 8H12 and 3E2, containing the constant regions of human IgG1, were subcloned into pTT5 expression vectors. For engineering Bis-83-1, the 8H12 and 3E2 scFv were linked with a GS(G_4_S)_4_ linker, and fused to the N-terminus of the human IgG1 Fc regions to generate Bis-83-2 heavy chain. The Bis-83-1 antibody was expressed by co-transfection of the modified heavy chain plasmids into Expi293F cells using PEI. For engineering Bis-83-2, the vH, and vL of 8H12 were linked with the (G_4_S)_3_ linker to construct the scFv, and fused to the C-terminus of 3E2 heavy chain with a (G_4_S)_3_ linker to generate Bis-83-2 heavy chain. The plasmids encoding the Bis-83-2 heavy chain and 3E2 light chain were transiently co-transfected into Expi293F cells at 1:1 ratio using PEI. The heavy chain and light chain variable regions of 8H12 and 3E2 were subcloned into the same vectors to express chimeric antibody heavy chain and light chain, respectively. The co-transfected Expi293F cells were cultured for 7 days and antibodies were purified from culture supernatants using protein A columns.

### Size-exclusive chromatography

C8H12, C3E2, Bis-83-1, and Bis-83-2 were subjected to HPLC (Waters; Milford, MA) analysis using a TSK Gel G3000PWXL 7.8 × 300-mm column (TOSOH) equilibrated in PBS, pH 7.4. The system flow rate was maintained at 0.5 mL/min and eluted proteins were detected at 280 nm.

### Pseudovirus neutralization assay (LV)

Neutralizing capacities of nAbs against ancestral strains of SARS-CoV, SARS-CoV-2 the variant strains of SARS-CoV-2 were tested based on lentiviral (LV) pseudotyping particles bearing the S proteins of the above strains, according to a previous report (Chang et al. 2021). In brief, lentiviral pseudovirions carrying the S proteins were produced by co-transfection of a lentiviral packaging plasmid (psPAX2, Addgene), a plasmid containing the S gene, and a green fluorescent protein (mNeonGreen) reporter vector (pLvEF1α-mNG, carrying EF1α promoter-driven mNeonGreen expressing cassette) into 293T cells. Mixtures of serially diluted nAbs and LV pseudotyping particle inoculum (0.5 TU/cell) were incubated for 1 h, and then transferred into 96-well optically clear-bottomed culture plates pre-seeded with H1299-ACE2hR cells (H1299 cells that stably over-expressing human ACE2 and nuclear-localized H2B-mRuby3) for 36 h. Fluorescence images were collected by Opera Phenix or Operetta CLS high-content equipment (PerkinElmer) and quantitatively determined using Columbus Software 2.5.0 (PerkinElmer). Antibody neutralizing activity was determined as a reduction in the percentage of mNeonGreen (+) cells in the nAb-treated wells as compared with the control wells. IC_50_ values were determined using the 4-parameter logistic (4PL) regression in GraphPad Prism (v8.0.1).

### Pseudovirus neutralization assay (VSV)

Pseudovirus of the SARS-CoV-2 prototyped strain was conducted based on VSV by carrying the S proteins as our previous study (Wang et al. 2023). Briefly, 2-fold serially diluted nAbs in 10% FBS-DMEM (Gibco, 12100061) from an initial concentration of 2 µg/mL, were mixed with diluted pseudovirus (MOI = 0.05) to a final volume of 80 µL and incubated at 37°C for 1 h. The mixture was then added to pre-seeded BHK21-hACE2 cells (BHK21 cells stably expressing hACE2) and incubated for 12 h. Post-infected cells were fluorescently imaged using Opera Phenix or Operetta CLS (PerkinElmer) and quantitatively analyzed by Columbus image management analysis software to detect the number of green fluorescent-positive cells. The inhibition rate was calculated as a reduction in the number of GFP-positive cells in the presence of the nAb(s) compared with untreated control cells. The IC_50_ values were identified as the maximum dilution concentration required to achieve infection inhibition by 50%, determined by the 4-parameter logistic (4PL) regression using GraphPad Prism (version 8.0.1).

### Live SARS-CoV-2 neutralization assay

SARS-CoV-2 live virus focus reduction neutralization test (FRNT) was performed in a certified Biosafety Level 3 laboratory, as previously described (Ju et al. 2020, Li et al. 2021b). Neutralization assays against live SARS-CoV-2 were conducted using a clinical isolate (EPI_ISL_406594, GWHBDSE01000000, B.1.351), previously obtained from a nasopharyngeal swab of an infected patient. Serial dilutions of tested antibodies were mixed with 50 µL of virus (100 focus forming units) in 96-well microwell plates and incubated at 37°C for 1 h. Mixtures were then transferred to 96-well plates seeded with Vero E6 cells and allowed to absorb for 1 h at 37°C. Inoculums were removed before adding the overlay media (100 µL MEM containing 1.6% carboxymethylcellulose). The plates were then incubated at 37°C for 24 h. Overlays were removed and cells were fixed with 4% paraformaldehyde solution for 30 min before permeabilization with Perm/Wash buffer (BD Biosciences) containing 0.1% Triton X-100 for 10 min. Cells were incubated with rabbit anti-SARS-CoV-2 NP IgG (Sino Biological, Inc.) for 1 h at room temperature followed by HRP-conjugated goat anti-rabbit IgG (H + L) antibody (TransGen Biotech, Beijing). The reactions were developed with KPL TrueBlue Peroxidase substrates (Seracare Life Sciences Inc.). SARS-CoV-2 foci were calculated using an EliSpot reader (Cellular Technology Ltd.).

### Enzyme-linked immunosorbent assay

SARS-CoV-2 spike trimer protein was coated into the wells of 96-well microplates at 100 ng per well and incubated overnight at 4°C. Plates were blocked with enzyme-linked immunosorbent assay (ELISA)-blocking buffer (Wantai BioPharm) and then incubated with 3-fold serially diluted antibodies diluted from a starting concentration of 10 µg/mL for 1 h at 37°C. Wells were then washed five times with PBST buffer [20 mmol/L PBS (pH 7.4), 150 mmol/L NaCl, and 0.05% Tween-20] and then incubated with HRP-conjugated anti-mouse IgG for 30 min at 37°C to detect the bound mAbs. Finally, the substrate solution was incubated for 15 min at 37°C, with the reaction quenched by the addition of 50 µL of 2 mol/L H_2_SO_4_. OD values were determined at 450 nm with a reference wavelength of 630 nm. The half-maximal effective concentration (EC_50_) was calculated by the 4-parameter logistic (4PL) regression using GraphPad Prism 8 software (version 8.0.1).

### Affinity determination by SPR

The binding affinities of the mAbs to different spike-trimer proteins or RBDs were determined by SPR assays using a Biacore 8K instrument (GE Healthcare). The viral antigen proteins (S2P/S6P or RBD) were covalently amine-coupled to CM5 and C1 sensor chips, respectively. Serially diluted antibodies (800, 400, 200, 100, 50, 25, 12.5, and 6.25 nmol/L) then flowed through the sensor surface at a flow rate of 30 µL/min in PBS-P+ buffer (0.2 mol/L phosphate buffer with 27 mmol/L KCl, 1.37 mol/L NaCl, and 0.5% Tween-20). The flow durations were 120 s for the association stage and 300 s for the dissociation stage. Finally, affinity constants (*K*_D_) were calculated using the evaluation software equipped for the Biacore 8K instrument.

For competitive SPR, mAbs or hACE2 were diluted to 1,600 nmol/L in PBS-P buffer. Antibodies (first protein) were loaded onto the biosensors for 180 s binding and then subjected to flow of a second interacting protein (hACE2 or the second antibody) for another 500 s. The unblocked pattern of the spike protein in buffer was used as a control.

### Cryo-EM sample preparation and data collection

Aliquots (3 µL) of 3.0 mg/mL mixtures of purified spike proteins of SARS-CoV-2 WT, BA.1, BA.2, BA.2.75 (Acro Biosystems), and BA.4/5 (Sino Biosystems) in complex with excess Fab fragments of single-, double-, or triple antibody combinations were incubated in 0.01% (*v*/*v*) Digitonin (Sigma) and then loaded onto glow-discharged (80 s at 20 mA) holey carbon Quantifoil grids (R1.2/1.3, 200 mesh, Quantifoil Micro Tools) using a Vitrobot Mark IV (Thermo Fisher Scientific) at 100% humidity and 4°C. As for the samples, WT-S complexed with single 8H12 and 3E2 were acquired using the EPU software on a Tecnai F30 TEM (Thermo Fisher Scientific) operated at 300 kV. Images were recorded using an FEI Falcon 3 detector in a 39-frame movie mode at a calibrated 93,000× magnification with an electron dose of 50 e^−^·Å^−2^. The remaining data were acquired using the SerialEM software on a Tecnai F30 TEM (Thermo Fisher Scientific) equipped with Gatan K3 direct detector. Images were recorded in 36-frame movie mode at a nominal 39,000× magnification at super-resolution mode with a pixel size of 0.389 Å, and the total electron dose was set to 60 e^−^·Å^−2^, with an exposure time was 4.5 s.

### Image processing and 3D reconstruction

Drift and beam-induced motion correction were performed with MotionCor2 (Zheng et al. 2017) to produce a micrograph from each movie. Contrast transfer function (CTF) fitting and phase-shift estimation were conducted with Gctf (Zhang 2016). Micrographs with astigmatism, obvious drift, or contamination were discarded before reconstruction. The following reconstruction procedures were performed using Cryosparc V3 (Punjani et al. 2017). In brief, particles were automatically picked using the “Blob picker” or “Template picker.” Several rounds of reference-free 2D classifications were performed and the selected “good” particles were then subjected to *ab initio* reconstruction, heterogeneous refinement, and final non-uniform refinement. Localized refinement focusing of the antibody interface were also performed if necessary. The resolutions of all density maps were determined by the gold-standard Fourier shell correlation curve, with a cutoff of 0.143 (Scheres and Chen 2012). Local resolutions of maps were estimated with ResMap (Kucukelbir et al. 2014).

### Atomic model building, refinement, and 3D visualization

The initial models of the nAbs were generated from homology modeling using Accelrys Discovery Studio software. The structure of RBD was taken from the structure of WT trimeric spike (pdb no. 6VSB (Wrapp et al. 2020) and used as the initial models of our WT-RBD and Omicron subvariants RBDs. We initially fitted the templates into the corresponding final cryo-EM maps using Chimera (Pettersen et al. 2004), and further corrected and adjusted them manually by real-space refinement in Coot (Emsley and Cowtan 2004). The resulting models were then refined with phenix.real_space_refine in PHENIX (Adams et al. 2010). These operations were executed iteratively until the problematic regions, Ramachandran outliers, and poor rotamers were either eliminated or moved to favored regions. The final atomic models were validated with Molprobity (Chen et al. 2010, Robert and Gouet 2014). All figures were generated with Chimera (Pettersen et al. 2004) or ChimeraX (Goddard et al. 2018, Pettersen et al. 2021).

### Statistical analysis

GraphPad Prism (version 8.0.1) was used for all statistical calculations. EC_50_ and IC_50_ values were calculated by non-linear regression analysis [log_(agonist)_ vs. response − variable slope (four parameters)].

## Supplementary Material

pwad040_suppl_Supplementary_Material

pwad040_suppl_Supplementary_Tabel_S2

## Data Availability

Structure coordinates are deposited in the Protein Data Bank under accession codes 8IV5 (WT-S:8H12:1C4-interface), 8IV8 (WT-S:3E2:1C4-interface), 8IV4, (WT-S:8H12:3E2-interface), and 8IVA (WT-S:XMA01:3E2-interface). The corresponding EM density maps have been deposited in the Electron Microscopy Data Bank under accession numbers EMD-35730 (WT-S:8H12), EMD-35731 (WT-S:3E2), EMD-35736 (WT-S:8H12:1C4), EMD-35741 (WT-S:8H12:1C4-interface), EMD-35739 (WT-S:3E2:1C4), EMD-35746 (WT-S:3E2:1C4-interface), EMD-35743 (WT-S:8H12:3E2), EMD-35740 (WT-S:8H12:3E2-interface), EMD-35749 (WT-S:XMA01:3E2), EMD-35755 (WT-S:XMA01:3E2-interface), EMD-35750 (WT-S:S2E12:3E2), EMD-35751 (WT-S:S2E12:3E2-interface), EMD-35745 (BA.1-S:8H12:3E2), EMD-35747 (BA.2-S:8H12:3E2) EMD-35752 (WT-S:8H12:S2X35), EMD-35754 (WT-S:8H12: S2X35-interface), and EMD-35753 (WT-S:8H12:VacW-209).

## Abbreviations

2D: two-dimensional
3D: three-dimensional
ACE2: angiotensin-converting enzyme 2
bnAbs: broad neutralizing antibodies
COVID-19: coronavirus disease of 2019
cryo-EM: cryo-electron microscopy
DMEM: Dulbecco’s modified Eagle’s medium
EC50: 50% effective concentration
ELISA: enzyme-linked immunosorbent assay
Fab: fragment of antigen binding
FBS: fetal bovine serum
FRNT: focus reduction neutralization test
FSC: Fourier shell correlation
IC_50_: 50% inhibition concentration
LV: lentivirus
mAbs: monoclonal antibodies
nAbs: neutralizing antibodies
PBS: phosphate-buffered saline
RBD: receptor-binding domain
RMSD: root Mean squared deviation
SARS-CoV: severe acute respiratory syndrome coronavirus
SARS-CoV-2: severe acute respiratory syndrome coronavirus 2
SPR: surface plasmon resonance
VOCs: variants of concern
VSV: vesicular stomatitis virus
