# Supplementary material for: Two antibodies show broad, synergistic neutralization against SARS-CoV-2 variants by inducing conformational change within the RBD
Source: Protein Cell. 2023 Jul 20;15(2):121–34. doi: 10.1093/procel/pwad040 (PMC10833452; doi:10.1093/procel/pwad040)
Supplement: pwad040_suppl_Supplementary_Material [file pwad040_suppl_supplementary_material.pdf]

Supplementary Information for

Two antibodies show broad, synergistic neutralization against SARS-CoV-2 variants by inducing conformational change within the RBD

Hui Sun[1, #], Tingting Deng[1, #], Yali Zhang[1,2, #], Yanling Lin[1, #], Yanan Jiang[1, #], Yichao Jiang[1, #],Yang Huang[1], Shuo Song[3], Lingyan Cui[1], Tingting Li[1,2], Hualong Xiong[1,2], Miaolin Lan[1], Liqin Liu[1], Yu Li[1], Qianjiao Fang[1], Kunyu Yu[1], Wenling Jiang[1], Lizhi Zhou[1,2], Yuqiong Que[1,2], Tianying Zhang[1,2], Quan Yuan[1,2], Tong Cheng[1,2], Zheng Zhang[3], Hai Yu[1,2], Jun Zhang[1,2], Wenxin Luo[1,2], Shaowei Li[1,2,*], Qingbing Zheng[1,2,*], Ying Gu[1,2,*], Ningshao Xia[1,2,4,*]

[1] State Key Laboratory of Molecular Vaccinology and Molecular Diagnostics; National Institute of Diagnostics and Vaccine Development in Infectious Diseases; State Key Laboratory of Vaccines for Infectious Diseases, School of Public Health, School of Life Sciences, Xiamen University, Xiamen 361102, China

[2] Xiang An Biomedicine Laboratory, Xiamen 361102, China

[3] Institute for Hepatology, National Clinical Research Center for Infectious Disease, Shenzhen Third People's Hospital; The Second Affiliated Hospital, School of Medicine, Southern University of Science and Technology, Shenzhen 518112, China

[4] Research Unit of Frontier Technology of Structural Vaccinology, Chinese Academy of Medical Sciences, Xiamen 361102, China

* Correspondence: shaowei@xmu.edu.cn (S.L.), abing0811@xmu.edu.cn (Q.Z.), guying@xmu.edu.cn (Y.G.) and nsxia@xmu.edu.cn (N.X.).

[#] These authors contributed equally.

This PDF file includes:

Supplementary Figs. S1-16.

Supplementary Tables S1-S2.

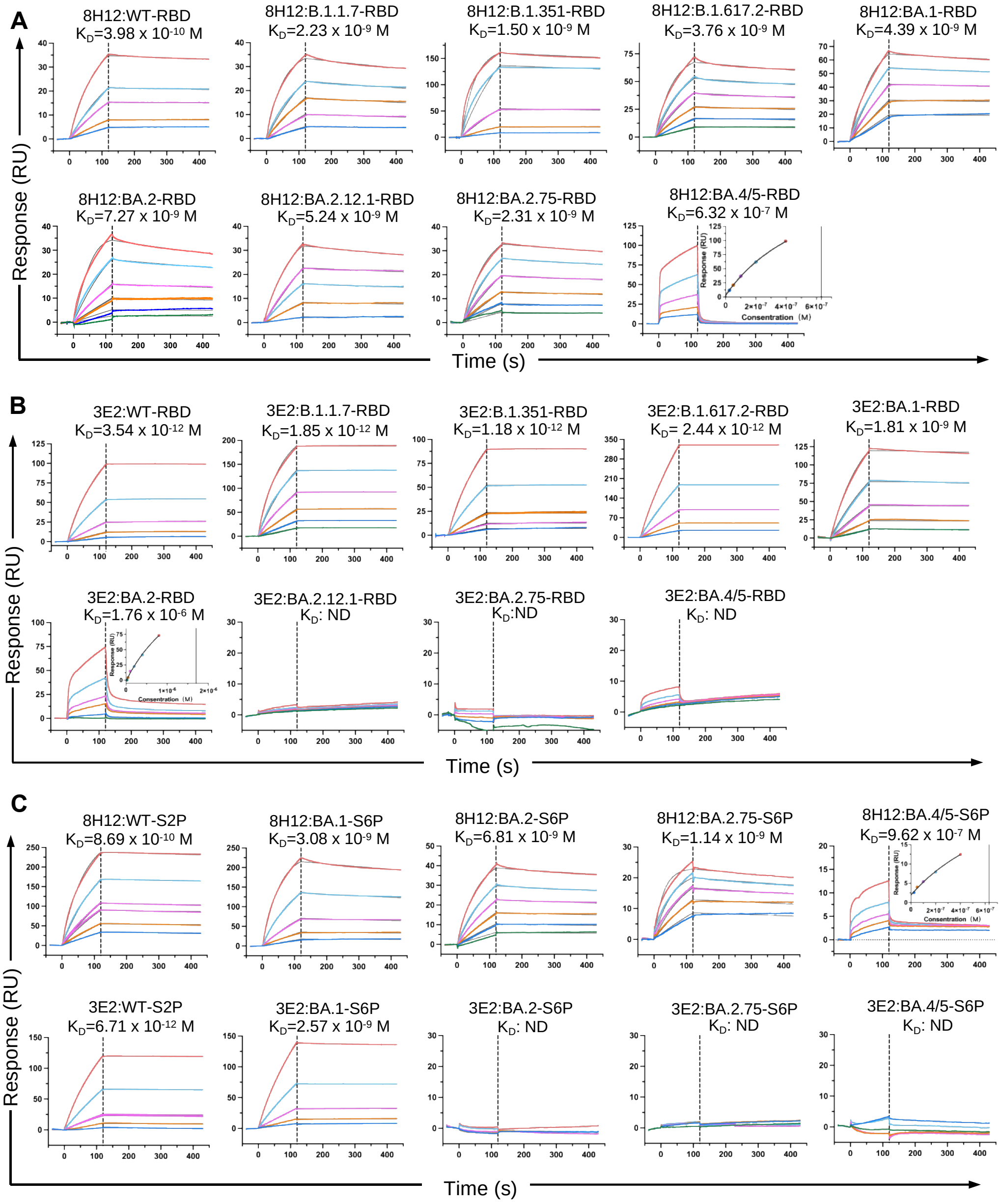

Fig. S1. Binding affinity identification of two antibodies against different antigens by SPR. (A-B) Binding curves of 8H12 (A) and 3E2 (B) to the SARS-CoV-2 RBDs including WT, Alpha, Beta, Delta, BA.1, BA.2, BA.2.12.1, BA.2.75, BA.4/5, respectively. **(C)** Binding curves of 8H12 and 3E2 to SARS-CoV-2 spike proteins including WT-S2P and Omicron subvariants S6P (BA.1-S6P, BA.2-S6P, BA.2.75-S6P and BA.4/5-S6P), respectively. Colored curves are the experimental traces obtained from surface plasmon resonance (SPR) experiments, and curves indicated the best

local fit for the data are used to calculate the KD values by using a 1:1 binding model or steady state affinity. ND indicates not detectable.

A

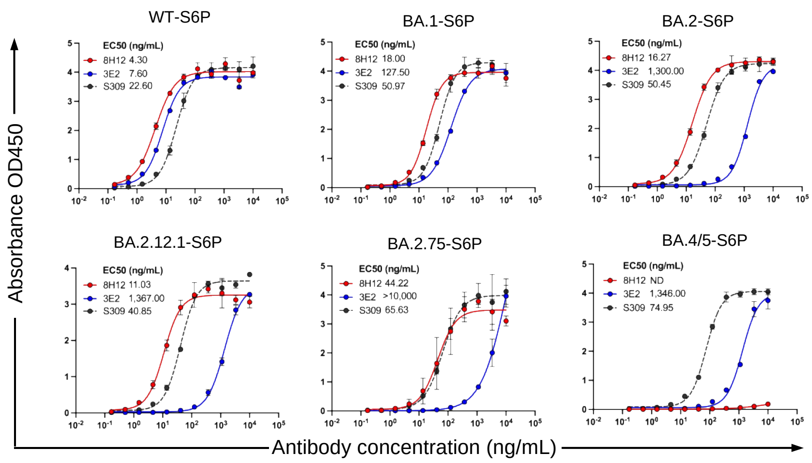

B

Summary of ELISA binding assays with EC_{50} (ng/mL) for 8H12 and 3E2

	WT-S6P	BA.1-S6P	BA.2-S6P	BA.2.12.1-S6P	BA.2.75-S6P	BA.4/5-S6P
**8H12**	4.30	18.00	16.27	11.03	44.22	ND
**3E2**	7.60	127.50	1,300.00	1,367.00	>10,000	1,346.00
**S309**	22.60	50.97	50.45	40.85	65.63	74.95

Fig. S2. Binding efficacies of the two antibodies against different antigens. (A) ELISA binding efficacies of 8H12 and 3E2 to trimeric S6P protein of WT and Omicron subvariants. S309 was selected as the positive control. Calculated EC50 values are shown. **(B)** Summary of the binding efficacies of three nAbs.

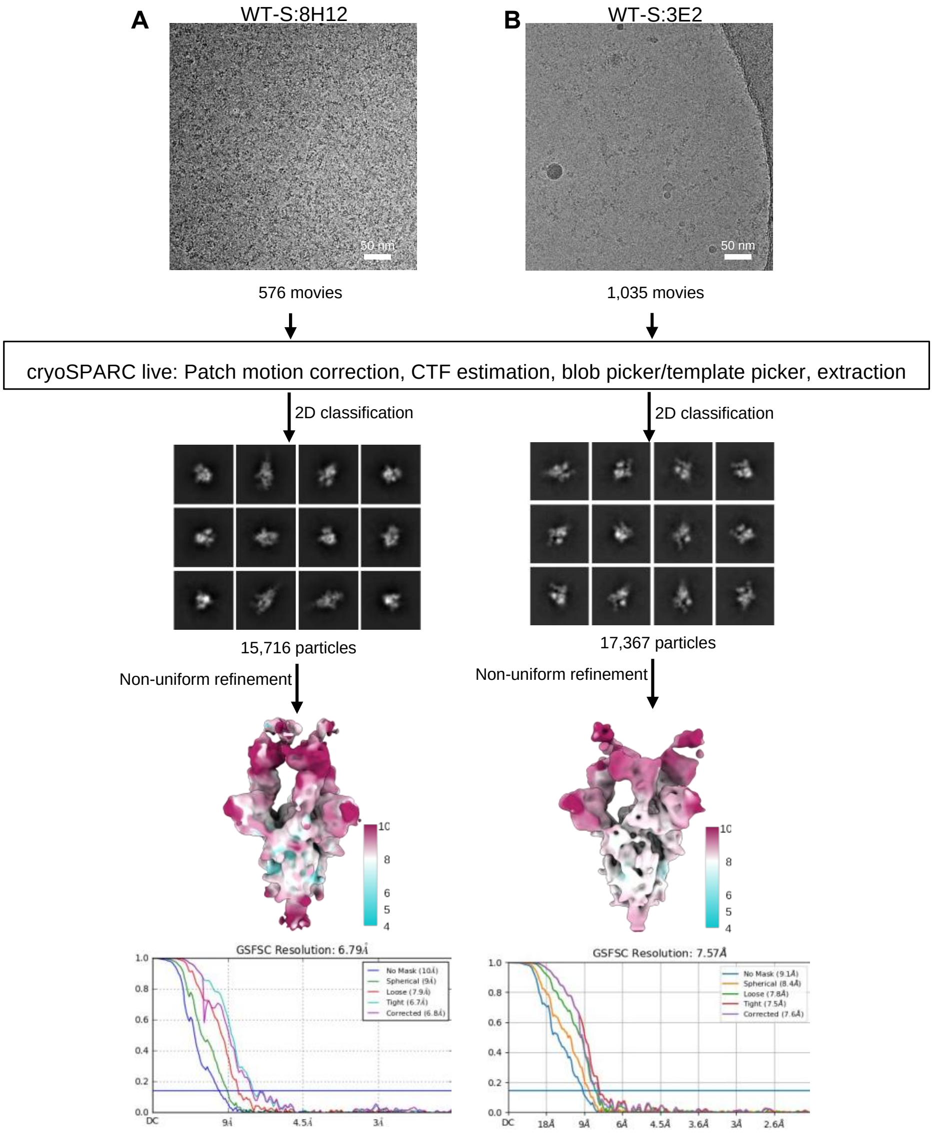

Fig. S3. Single-particle cryo-EM image processing workflow for the mono-antibody immune-complexes. (A-B) Flowcharts for cryo-EM analysis of immune complexes of WT-S:8H12 (A) and WT-S:3E2 (B). Representative electron micrographs, 2D classifications, heterogeneous refinement maps, final maps colored by local resolution and FSC curves are shown. Scale bar: 50nm.

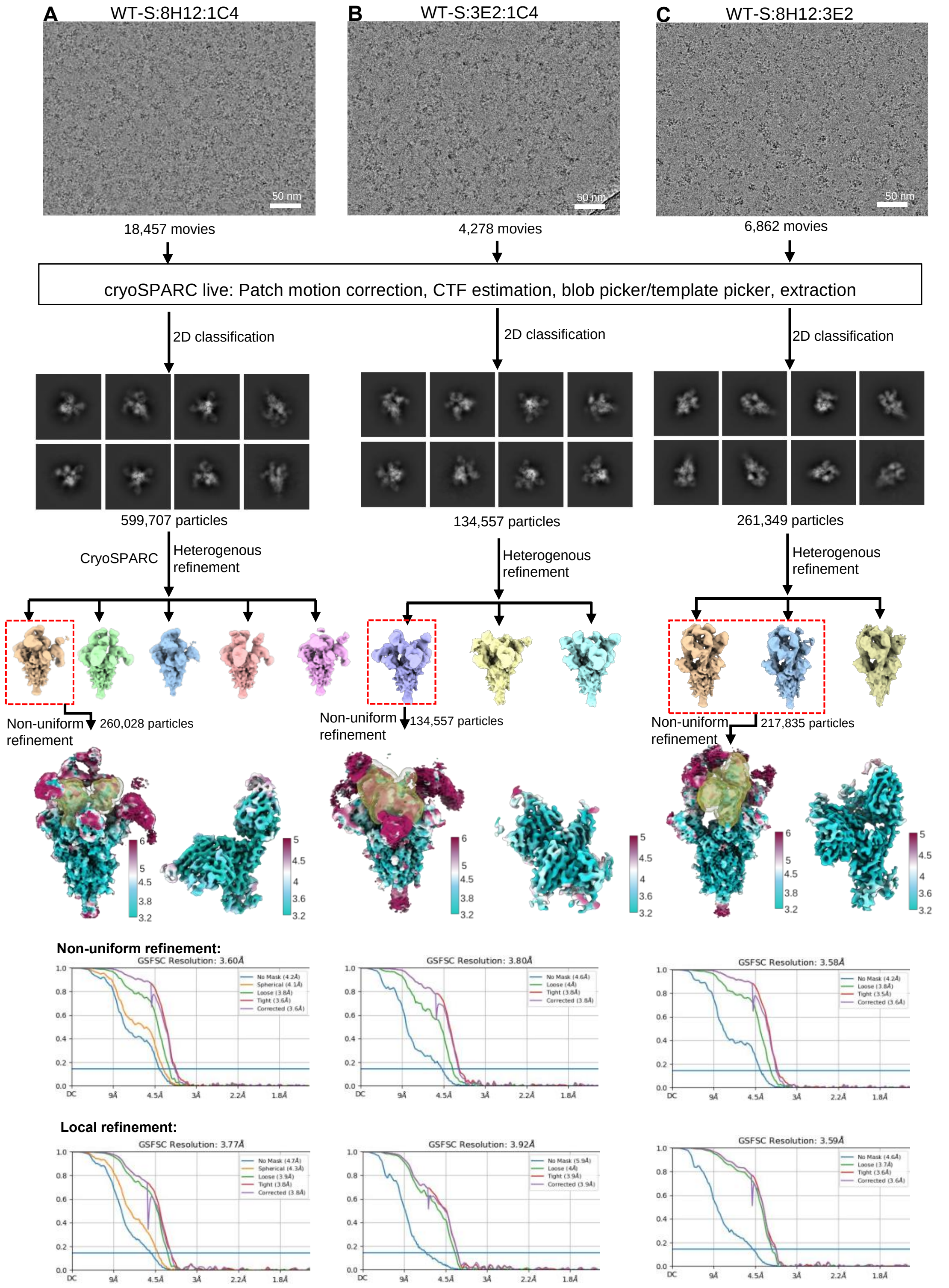

Fig. S4. Single-particle cryo-EM image processing workflow for the double-antibody immune-complexes. (A-C) Flowcharts for cryo-EM analysis of immune complexes of WT-S:8H12:1C4 (A), WT-S:3E3:1C4 (B) and WT-S:8H12:3E2

(C). Representative electron micrographs, 2D classifications, heterogeneous refinement maps, final maps colored by local resolution and FSC curves are shown. Scale bar: 50 nm.

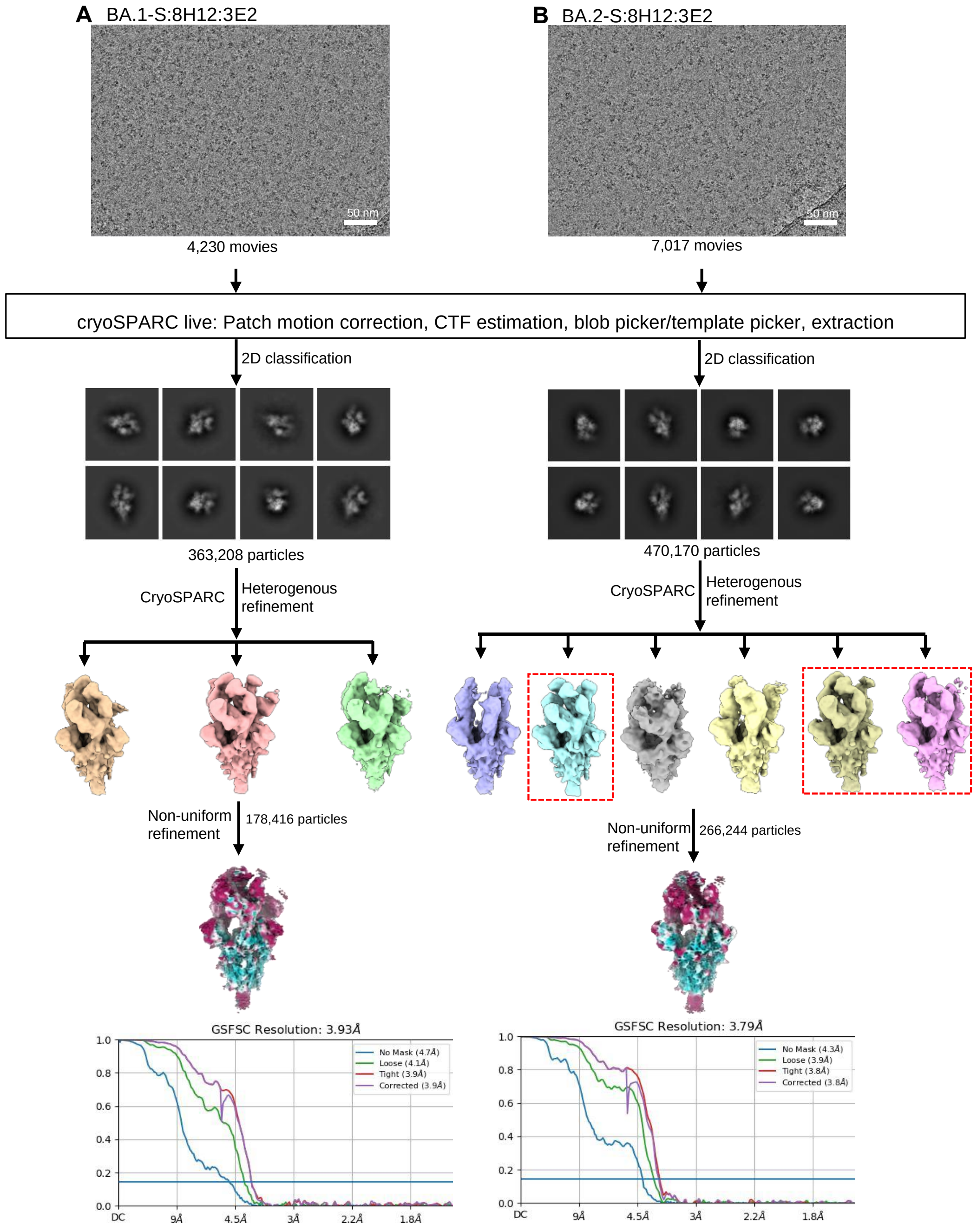

Fig. S5. Single-particle cryo-EM image processing workflow for the immune-complexes of BA.1-S:8H12:3E2 and BA.2-S:8H12:3E2. (A-B) Flowcharts for cryo-EM analysis of immune complexes BA.1-S:8H12:3E2 (A) and BA.2-S:8H12:3E2 (B). Representative electron micrographs, 2D classifications, heterogeneous refinement maps, final maps colored by local resolution and FSC curves are shown. Scale bar: 50 nm.

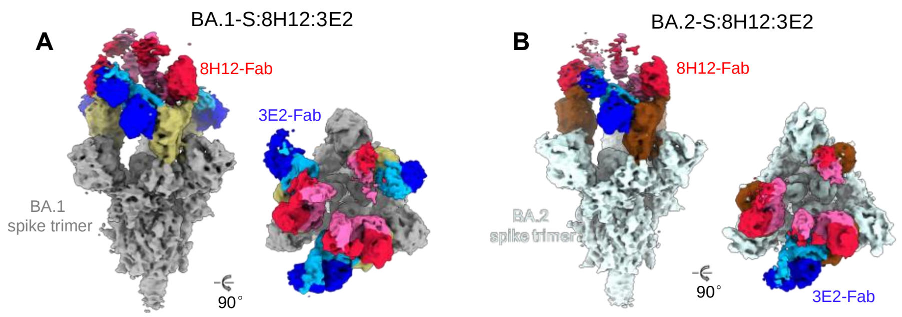

Fig. S6. Cryo-EM structures of 8H12/3E2 double-antibody in complex with spike proteins of Omicron BA.1 and BA.2. (A-B) Domain-colored cryo-EM density maps of the immune complexes of BA.1-S:8H12:3E2 (A) and BA.2-S:8H12:3E2 (B) are shown.

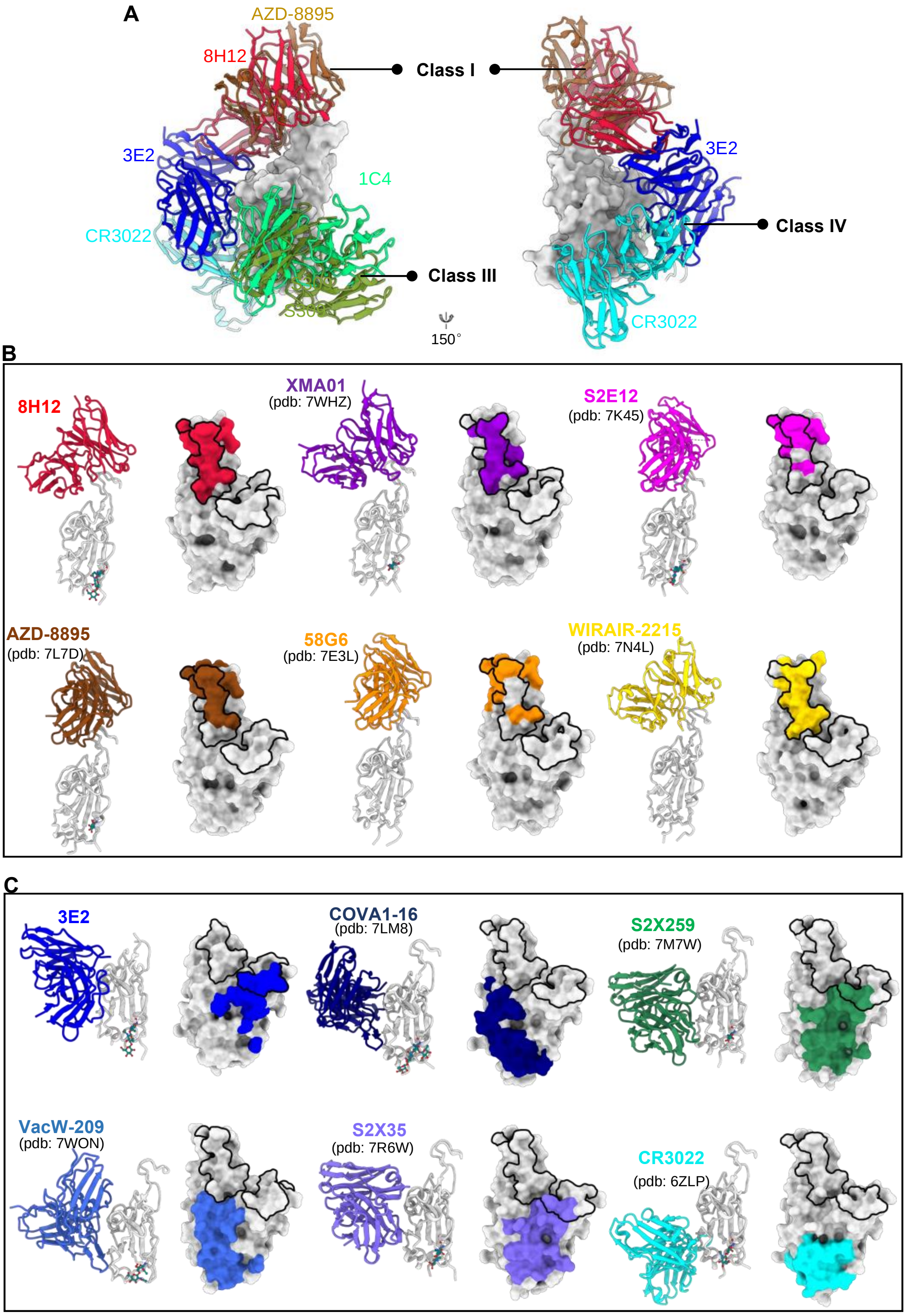

Fig. S7. Classification and comparison of RBD-specific antibodies with diverse binding modes. (A) Superimposition of three nAbs in this study to the representative Class I-IV nAbs. **(B)** Comparison of the footprints of 8H12 and 8H12-like nAbs. **(C)**

Comparison of the footprints of 3E2 and 3E2-like nAbs.

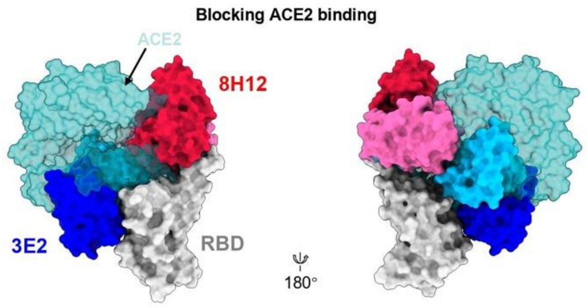

Fig. S8. Superimposition of structures of ACE2-RBD complex (pdb no. 7C8D) and WT-RBD:8H12:3E2. Potent steric clashes between ACE2 and both antibodies are observed, which indicates the two nAbs could block the binding of ACE2 to the RBD.

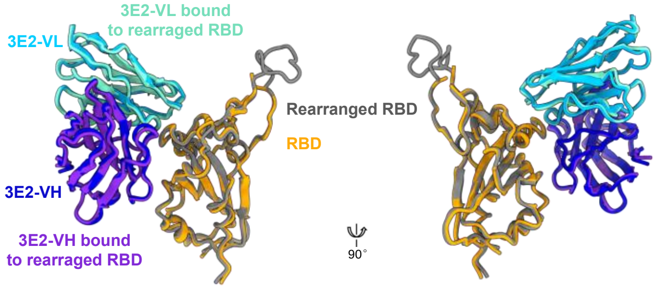

Fig. S9. Superimposition of structures of WT-RBD:3E2 (derived from the structure WT-S:3E2:1C4) and rearranged-RBD:3E2 (derived from the structure WT-S:8H12:3E2). The 3E2 binding orientation remain unchanged in the structure WT-S:8H12:3E2.

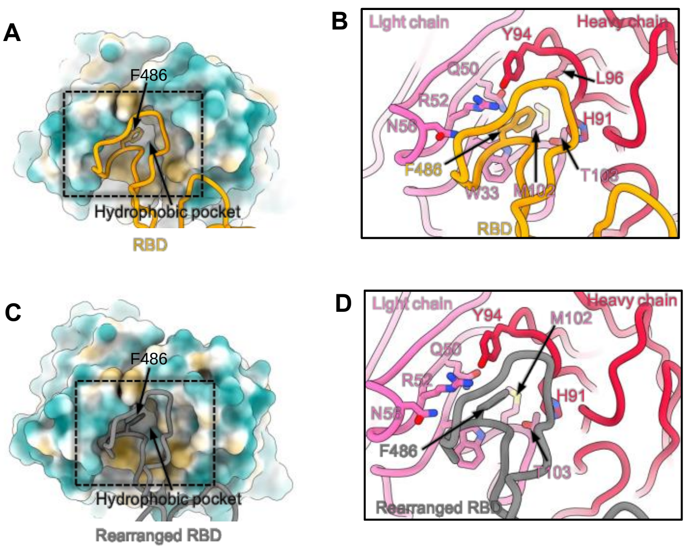

Fig. S10. The critical role of the RBD F486 in the interaction with 8H12. (A and C) the F486 interacts with 8H12 by inserting itself into a hydrophobic pocket formed by 8H12-VH and 8H12-VL, which is observed in both the structures of WT-RBD:8H12:1C4 (A) and WT-RBD:8H12:3E2 (C). The 8H12 is presented as surface colored with molecular lipophilicity potential (cyan, hydrophilic; white, neutral; goldenrod, hydrophobic) and the RBD is presented as cartoon. **(B and D)** Detailed views showing the F486 mediated interactions. The contact residues of 8H12 involved in contact interaction are labaled and represented as stick.

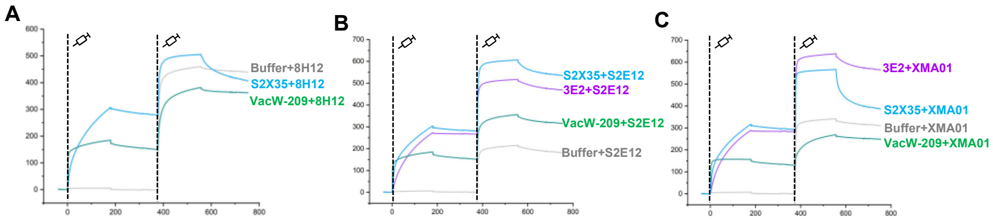

Fig. S11. Simultaneously binding potencies of 8H12-like and 3E2-like double-antibody combinations. (A-C) Competition SPR sensorgrams of double-antibody combinations. The combinations of 8H12 and 3E2-like mAbs (A), 8H12-like S2E12 and 3E2/3E2-like mAbs (B), 8H12-like XMA01 and 3E2/3E2-like mAbs (C) were tested, the results indicate their collaborative binding potencies. The mAbs were injected onto the WT-S6P-immobilized CM5 sensor chip. The unblocked pattern of the WT-S6P with buffer was used as control.

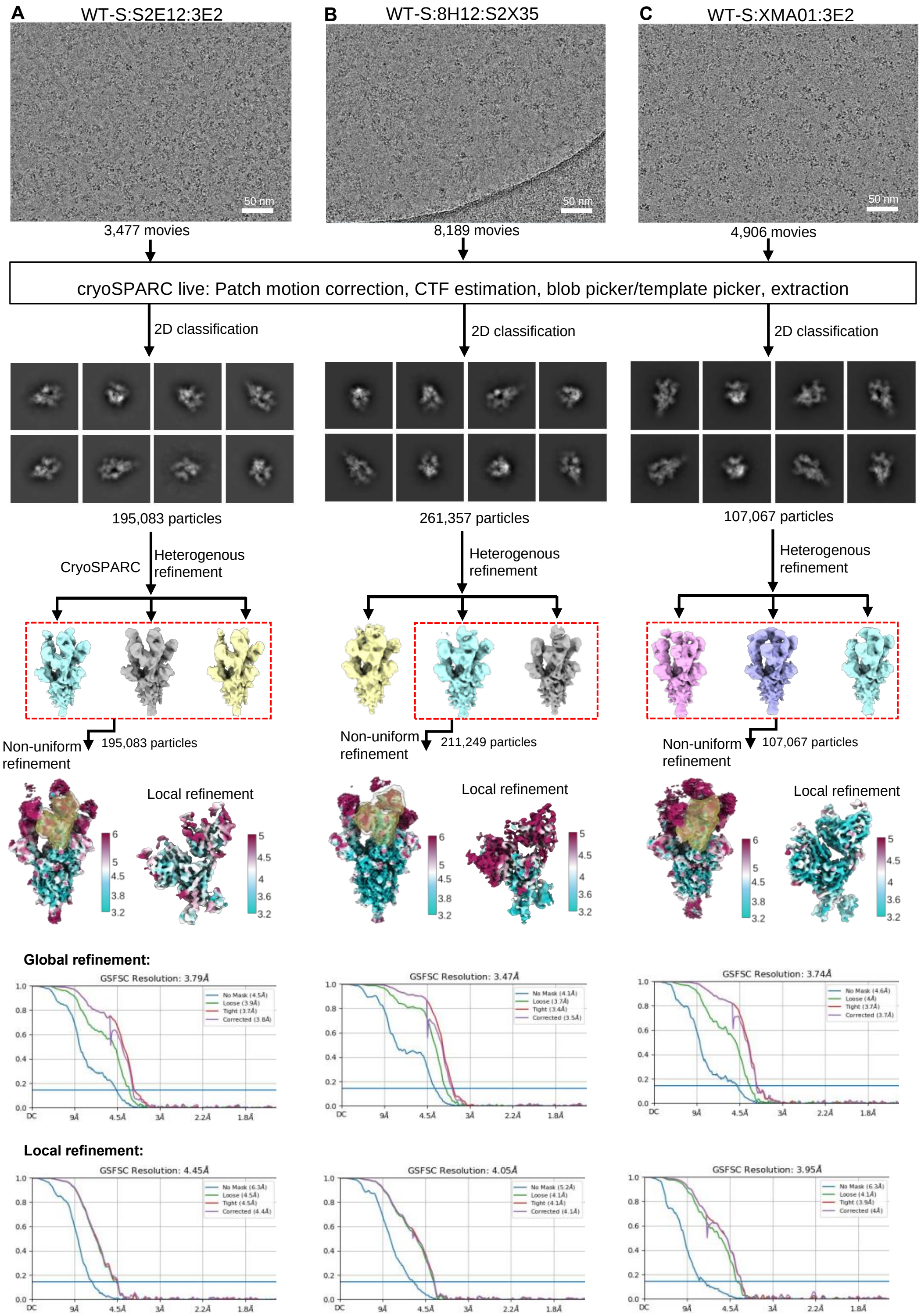

Fig. S12. Single-particle cryo-EM image processing workflow for the 8H12-like and 3E2-like double-antibody immune-complexes. (A-C) Flowcharts for cryo-EM analysis of immune complexes of WT-S:S2E12:3E2 (A), WT-S:8H12:S2X35 (B) and

WT-S:XMA01:3E2 (C). Representative electron micrographs, 2D classifications, heterogeneous refinement maps, final maps colored by local resolution and FSC curves are shown. Scale bar: 50 nm.

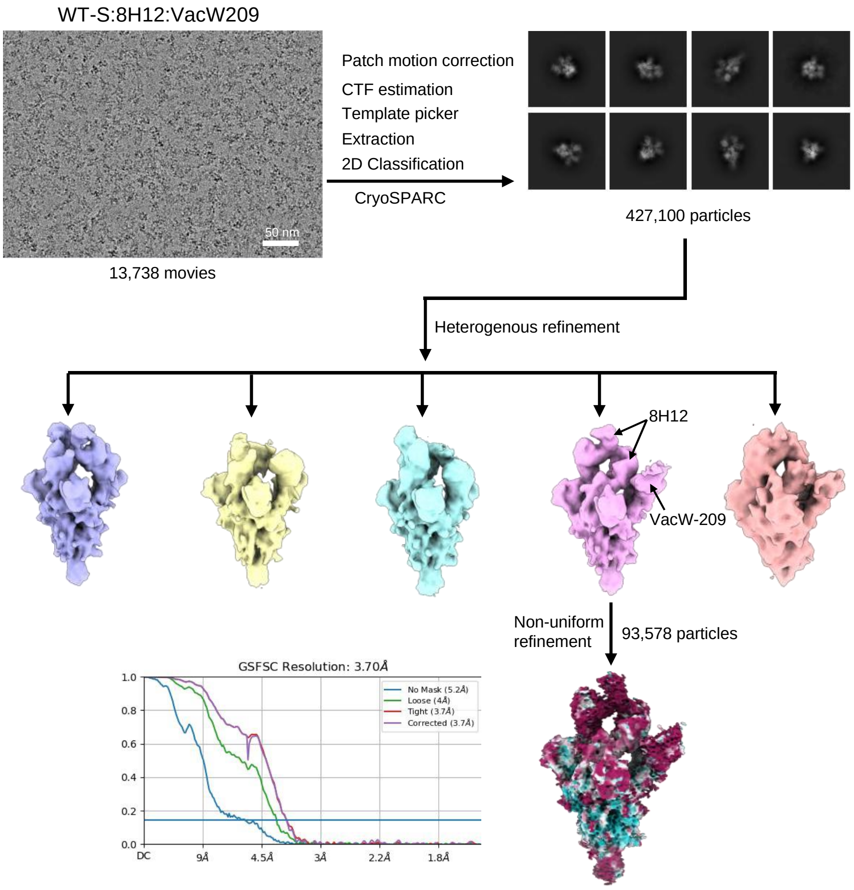

Fig. S13. Single-particle cryo-EM image processing workflow for S-2P in complex with 8H12 and 3E2-like mAb VacW-209. Representative electron micrographs, 2D classifications, heterogeneous refinement map, final map colored by local resolution and FSC curve are shown. Scale bar: 50 nm.

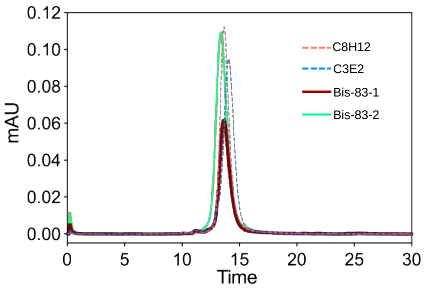

Fig. S14. Characterization of purified bispecific antibodies by HPLC analysis.

The parental mAbs C8H12 and C3E2 are included as control.

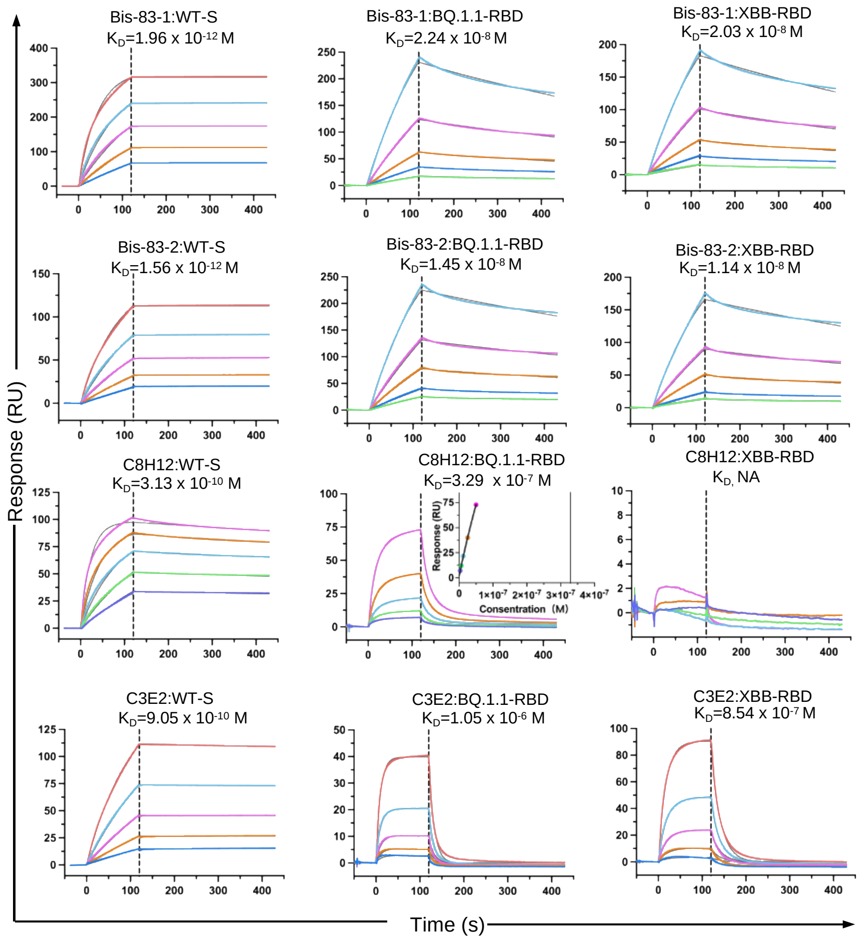

Fig. S15. Binding affinity identification of bispecific antibodies against different antigens by SPR. Binding curves of bispecific antibodies to the SARS-CoV-2 spike/RBDs including WT, BQ.1.1, XBB subvariants. Two human-murine chimeric nAb C8H12 and C3E2 are included as comparator control. Colored curves are the experimental traces obtained from surface plasmon resonance (SPR) experiments, and curves indicate the best local fit for the data are used to calculate the KD values by using a 1:1 binding model or steady state affinity. ND indicates not detectable.

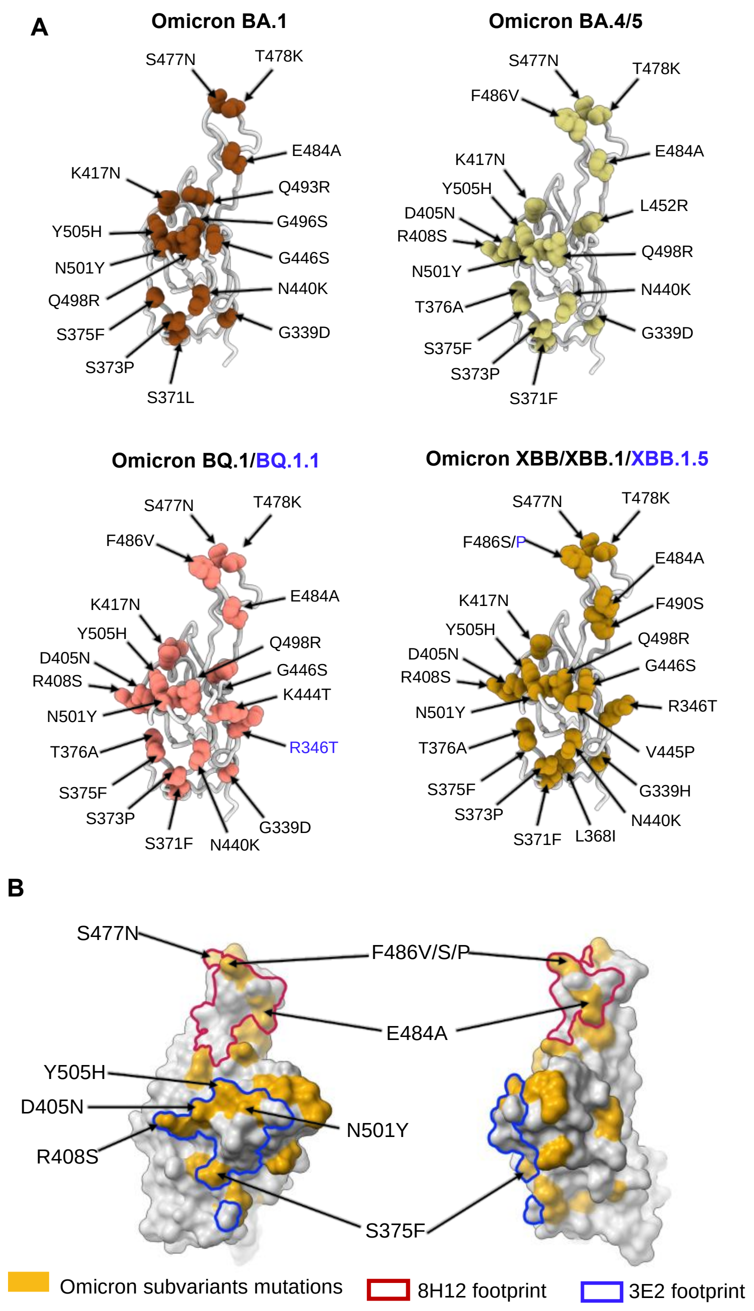

Fig. S16. Variant mutations distribution on the structures of the spike proteins of Omicron subvariants. (A) RBD (gray cartoon representation) mutations on the BA.1, BA.4/5, BQ.1/BQ.1.1, XBB/XBB.1/XBB.1.5 are represented by stick. **(B)** Binding footprints of 8H12 and 3E2 on the RBD. The residues involved in mutations on the Omicron (and its subvariants) RBDs are labelled.

Table S1. Neutralizing efficacies of 8H12, 3E2 and their combination

SARS-CoV-2 strains	IC50 (ng/mL)			
	8H12	3E2	8H12+3E2	S309
D614G	34	160	9	57
B.1.1.7	41	151	8	165
B.1.351	78	152	21	39
P.1	92	140	6	11
B.1.617.2	51	219	17	48
B.1.429	30	185	6	22
B.1.525	100	231	18	17
B.1.526a	44	153	13	49
B.1.526b	113	263	40	43
B.1.617.1	83	347	22	71
A.VOI.V2	137	355	44	25
C.37	27	145	15	28
B.1.529	163	280	119	214
BA.2	175	>45,000	244	1,271
BA.2.12.1	34	>45,000	509	1,170
BA.2.75	18	>45,000	35	275
BA.4	>45,000	>45,000	>45,000	1,023
BA.5	>45,000	>45,000	>45,000	636

IC50 (ng/mL): <10 | <100 | <1,000 | <10,000 | >45,000

Table S2. Cryo-EM data collection, refinement and validation statistics of immune-complexes

	WT-S:8H12	WT-S:3E2	WT-S:8H12:1C4	WT-S:8H12:1C4-interface	WT-S:3E2:1C4	WT-S:3E2:1C4-interface	WT-S:8H12:3E2	WT-S:8H12:3E2-interface	BA.1-S:8H12:3E2	BA.2-S:8H12:3E2	WT-S:S2E12:3E2	WT-S:S2E12:3E2-interface	WT-S:8H12:S2X35	WT-S:8H12:S2X35-interface	WT-S:XMA01:3E2	WT-S:XMA01:3E2-interface	WT-S:8H12:VacW-209
**Data Collection and processing**																	
Microscope	Tecnai F30	Tecnai F30	Tecnai F30	Tecnai F30	Tecnai F30	Tecnai F30	Tecnai F30	Tecnai F30	Tecnai F30	Tecnai F30	Tecnai F30	Tecnai F30	Tecnai F30	Tecnai F30	Tecnai F30	Tecnai F30	Tecnai F30
Camera	Falcon 3	Falcon 3	K3	K3	K3	K3	K3	K3	K3	K3	K3	K3	K3	K3	K3	K3	K3
Voltage (kV)	300	300	300	300	300	300	300	300	300	300	300	300	300	300	300	300	300
Electron exposure dose ($e^-/Å^2$)	50	50	60	60	60	60	60	60	60	60	60	60	60	60	60	60	60
Defocus range (μm)	1.0-2.4	1.0-2.4	0.9-1.8	0.9-1.8	1.0-2.2	1.0-2.2	1.0-2.4	1.0-2.4	1.0-2.0	1.0-2.2	0.9-1.8	0.9-1.8	1.0-2.2	1.0-2.2	1.1-2.0	1.1-2.0	1.0-1.8
Pixel size (Å)	1.12	1.12	0.778	0.778	0.778	0.778	0.778	0.778	0.778	0.778	0.778	0.778	0.778	0.778	0.778	0.778	0.778
Micrographs (used)	576	1,035	18,457	/	4,278	/	6,862	/	4230	7071	3,477	/	8,189	/	4,906	/	13,738
Final particle images (nos.)	15,716	17,367	260,028	260,028	134,557	107,067	217,835	217,835	178,416	266,244	195,083	195,083	211,249	211,249	107,067	107,067	93,578
Symmetry imposed	C3	C3	C1	C1	C1	C1	C1	C1	C1	C1	C1	C1	C1	C1	C1	C1	C1
Map resolution (Å)	6.79	7.57	3.60	3.77	3.80	3.92	3.58	3.59	3.93	3.79	3.79	4.45	3.47	4.05	3.74	3.95	3.70
FSC threshold	0.143	0.143	0.143	0.143	0.143	0.143	0.143	0.143	0.143	0.143	0.143	0.143	0.143	0.143	0.143	0.143	0.143
Map sharpening B factor ($Å^2$)	-440.3	-722.8	-98.6	-125.8	-91.5	-118.3	-89.9	-105.6	-94.5	-94	-95.0	-210.7	-96.2	-158.0	-79.6	-108.3	-60.6
**Validation**																	
MolProbity score	/	/	/	1.75	/	1.69	/	1.54	/	/	/	/	/	/	/	1.76	/
Poor rotamers (%)	/	/	/	0.91	/	0.74	/	0.55	/	/	/	/	/	/	/	0.54	/
Clashscore	/	/	/	5.11	/	5.17	/	3.15	/	/	/	/	/	/	/	5.49	/
RMS (bonds)	/	/	/	0.0072	/	0.0051	/	0.0057	/	/	/	/	/	/	/	0.0057	/
RMS (angles)	/	/	/	1.13	/	1.09	/	1.1	/	/	/	/	/	/	/	1.2	/
Ramachandran plot																	
Favored (%)	/	/	/	92.13	/	93.85	/	93.22	/	/	/	/	/	/	/	92.63	/
Allowed (%)	/	/	/	7.87	/	6.15	/	6.78	/	/	/	/	/	/	/	7.37	/
Disallowed (%)	/	/	/	0	/	0	/	0	/	/	/	/	/	/	/	0	/

Fig. S1

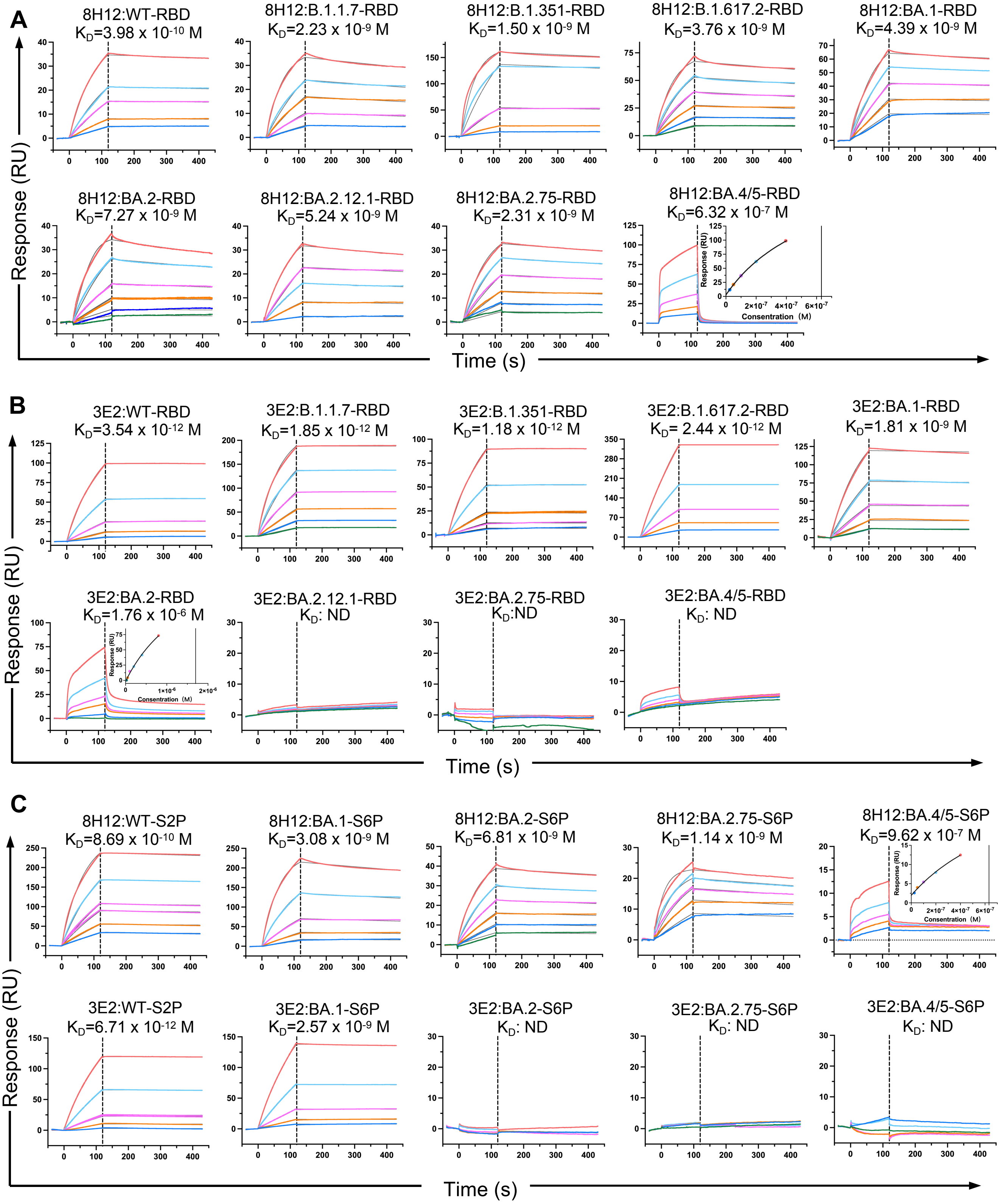
A
8H12:WT-RBD
KD=3.98 x 10-10 M
8H12:B.1.1.7-RBD
KD=2.23 x 10-9 M
8H12:B.1.351-RBD
KD=1.50 x 10-9 M
8H12:B.1.617.2-RBD
KD=3.76 x 10-9 M
8H12:BA.1-RBD
KD=4.39 x 10-9 M
8H12:BA.2-RBD
KD=7.27 x 10-9 M
8H12:BA.2.12.1-RBD
KD=5.24 x 10-9 M
8H12:BA.2.75-RBD
KD=2.31 x 10-9 M
8H12:BA.4/5-RBD
KD=6.32 x 10-7 M
Response (RU)
Consentration (M)
Response (RU)
Time (s)
B
3E2:WT-RBD
KD=3.54 x 10-12 M
3E2:B.1.1.7-RBD
KD=1.85 x 10-12 M
3E2:B.1.351-RBD
KD=1.18 x 10-12 M
3E2:B.1.617.2-RBD
KD= 2.44 x 10-12 M
3E2:BA.1-RBD
KD=1.81 x 10-9 M
3E2:BA.2-RBD
KD=1.76 x 10-6 M
3E2:BA.2.12.1-RBD
KD: ND
3E2:BA.2.75-RBD
KD:ND
3E2:BA.4/5-RBD
KD: ND
Response (RU)
Time (s)
C
8H12:WT-S2P
KD=8.69 x 10-10 M
8H12:BA.1-S6P
KD=3.08 x 10-9 M
8H12:BA.2-S6P
KD=6.81 x 10-9 M
8H12:BA.2.75-S6P
KD=1.14 x 10-9 M
8H12:BA.4/5-S6P
KD=9.62 x 10-7 M
3E2:WT-S2P
KD=6.71 x 10-12 M
3E2:BA.1-S6P
KD=2.57 x 10-9 M
3E2:BA.2-S6P
KD: ND
3E2:BA.2.75-S6P
KD: ND
3E2:BA.4/5-S6P
KD: ND
Response (RU)
Time (s)

Fig. S2

A

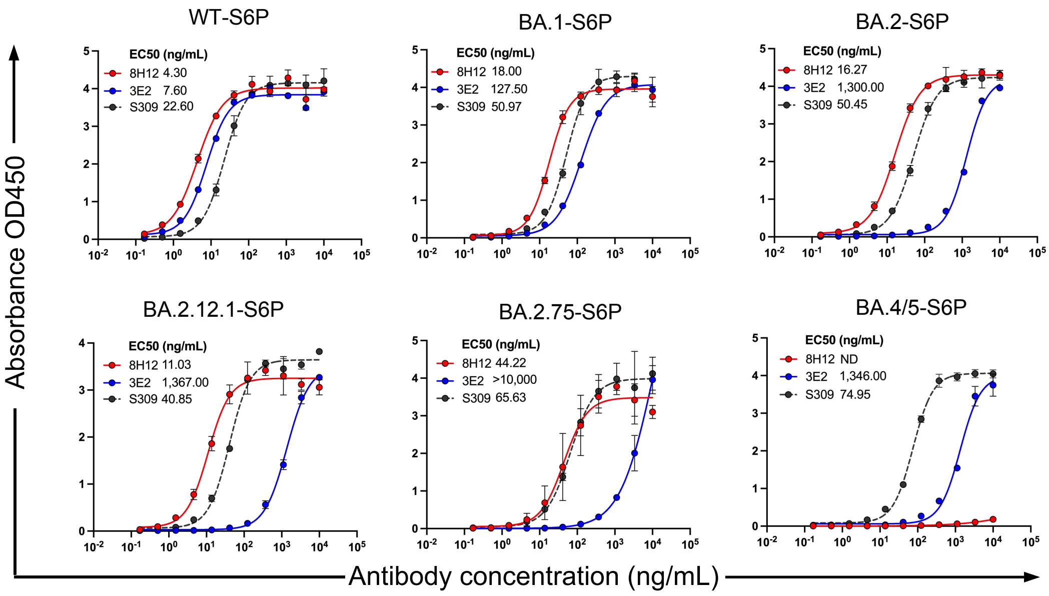

B

Summary of ELISA binding assays with EC_{50} (ng/mL) for 8H12 and 3E2

	WT-S6P	BA.1-S6P	BA.2-S6P	BA.2.12.1-S6P	BA.2.75-S6P	BA.4/5-S6P
**8H12**	4.30	18.00	16.27	11.03	44.22	ND
**3E2**	7.60	127.50	1,300.00	1,367.00	>10,000	1,346.00
**S309**	22.60	50.97	50.45	40.85	65.63	74.95

Fig. S3

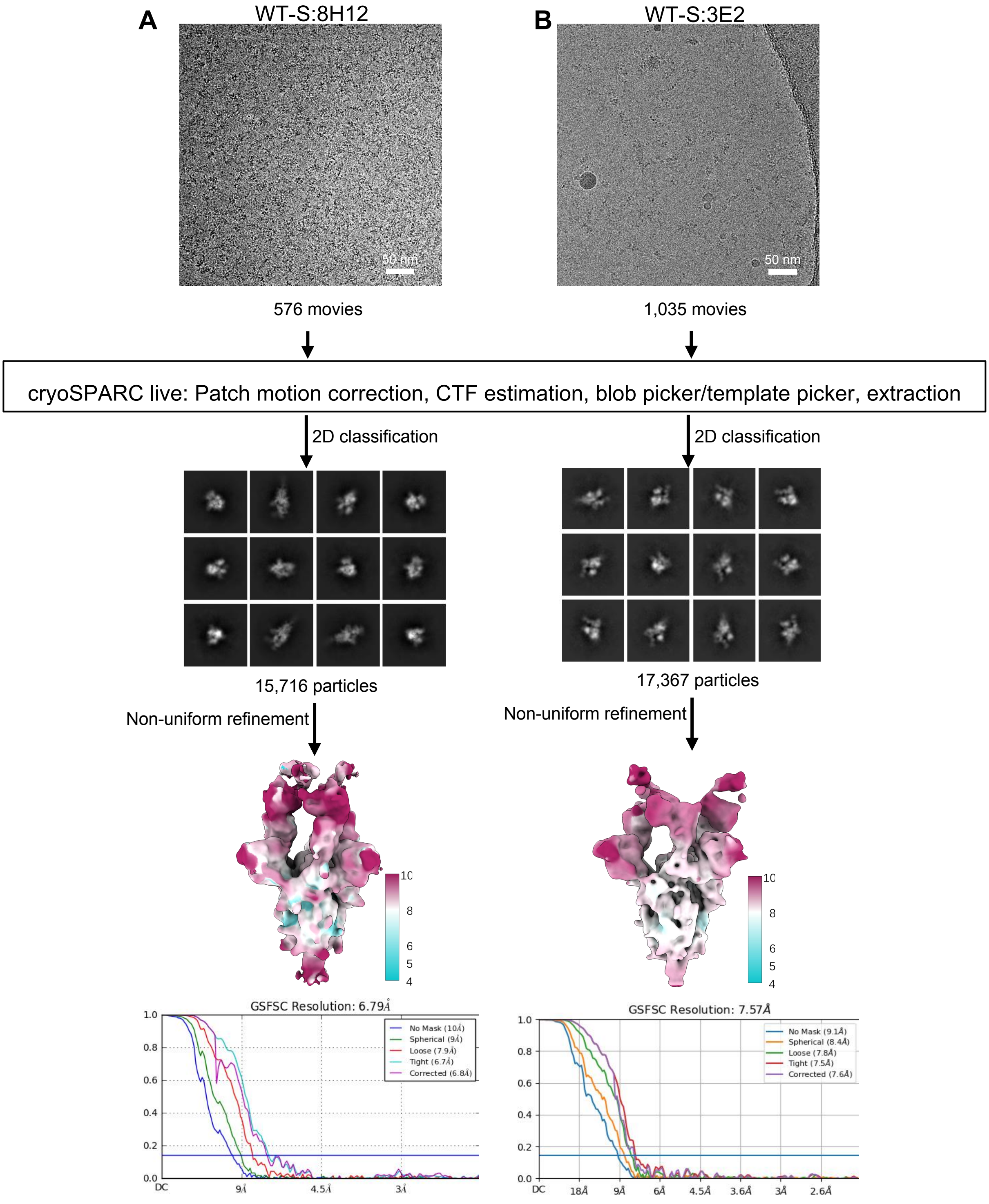

A
WT-S:8H12
50 nm
576 movies
B
WT-S:3E2
50 nm
1,035 movies
cryoSPARC live: Patch motion correction, CTF estimation, blob picker/template picker, extraction
2D classification
2D classification
15,716 particles
17,367 particles
Non-uniform refinement
Non-uniform refinement
GSFSC Resolution: 6.79Å
No Mask (10Å)
Spherical (9Å)
Loose (7.9Å)
Tight (6.7Å)
Corrected (6.8Å)
GSFSC Resolution: 7.57Å
No Mask (9.1Å)
Spherical (8.4Å)
Loose (7.8Å)
Tight (7.5Å)
Corrected (7.6Å)

Fig. S4

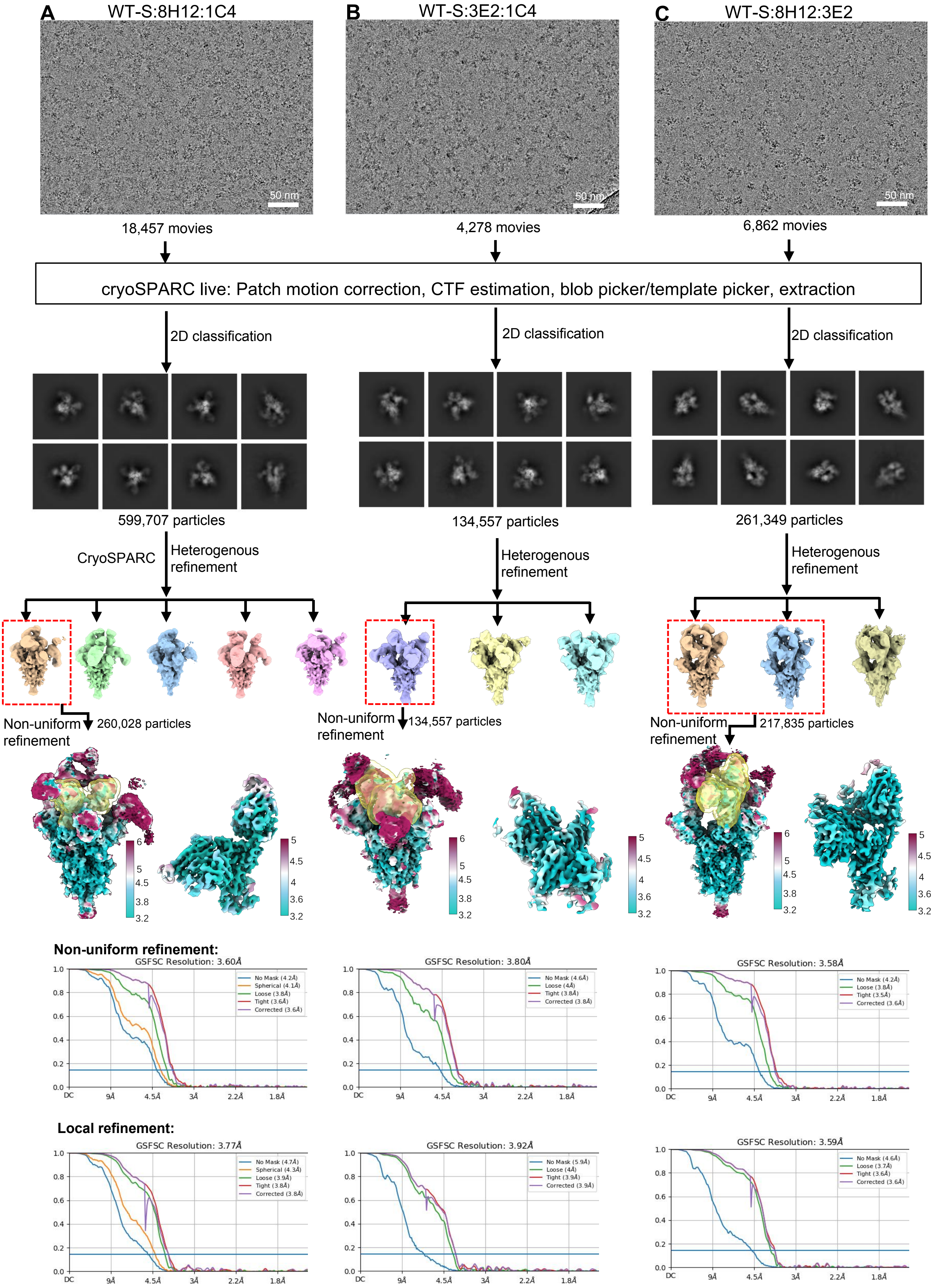
A
WT-S:8H12:1C4
B
WT-S:3E2:1C4
C
WT-S:8H12:3E2
50 nm
18,457 movies
4,278 movies
6,862 movies
cryoSPARC live: Patch motion correction, CTF estimation, blob picker/template picker, extraction
2D classification
599,707 particles
134,557 particles
261,349 particles
CryoSPARC
Heterogenous refinement
Non-uniform refinement
260,028 particles
134,557 particles
217,835 particles
Non-uniform refinement:
GSFSC Resolution: 3.60Å
No Mask (4.2Å)
Spherical (4.1Å)
Loose (3.8Å)
Tight (3.6Å)
Corrected (3.6Å)
GSFSC Resolution: 3.80Å
No Mask (4.6Å)
Loose (4Å)
Tight (3.8Å)
Corrected (3.8Å)
GSFSC Resolution: 3.58Å
No Mask (4.2Å)
Loose (3.8Å)
Tight (3.5Å)
Corrected (3.6Å)
Local refinement:
GSFSC Resolution: 3.77Å
No Mask (4.7Å)
Spherical (4.3Å)
Loose (3.9Å)
Tight (3.8Å)
Corrected (3.8Å)
GSFSC Resolution: 3.92Å
No Mask (5.9Å)
Loose (4Å)
Tight (3.9Å)
Corrected (3.9Å)
GSFSC Resolution: 3.59Å
No Mask (4.6Å)
Loose (3.7Å)
Tight (3.6Å)
Corrected (3.6Å)
DC
9Å
4.5Å
3Å
2.2Å
1.8Å

Fig. S5

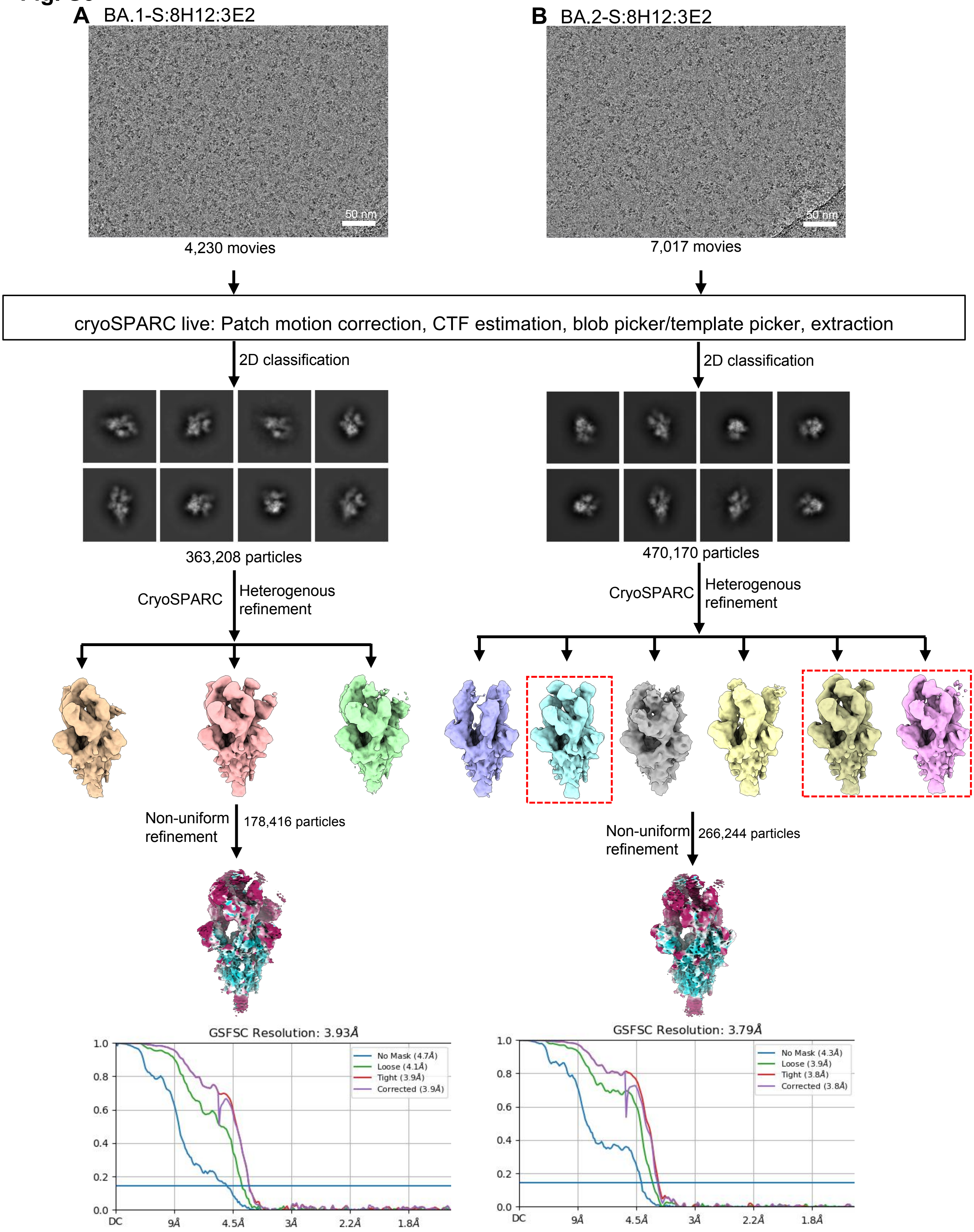

A BA.1-S:8H12:3E2
50 nm
4,230 movies
B BA.2-S:8H12:3E2
50 nm
7,017 movies
cryoSPARC live: Patch motion correction, CTF estimation, blob picker/template picker, extraction
2D classification
363,208 particles
CryoSPARC
Heterogenous refinement
Non-uniform refinement
178,416 particles
2D classification
470,170 particles
CryoSPARC
Heterogenous refinement
Non-uniform refinement
266,244 particles
GSFSC Resolution: 3.93Å
No Mask (4.7Å)
Loose (4.1Å)
Tight (3.9Å)
Corrected (3.9Å)
GSFSC Resolution: 3.79Å
No Mask (4.3Å)
Loose (3.9Å)
Tight (3.8Å)
Corrected (3.8Å)
1.0
0.8
0.6
0.4
0.2
0.0
DC
9Å
4.5Å
3Å
2.2Å
1.8Å

Fig. S6

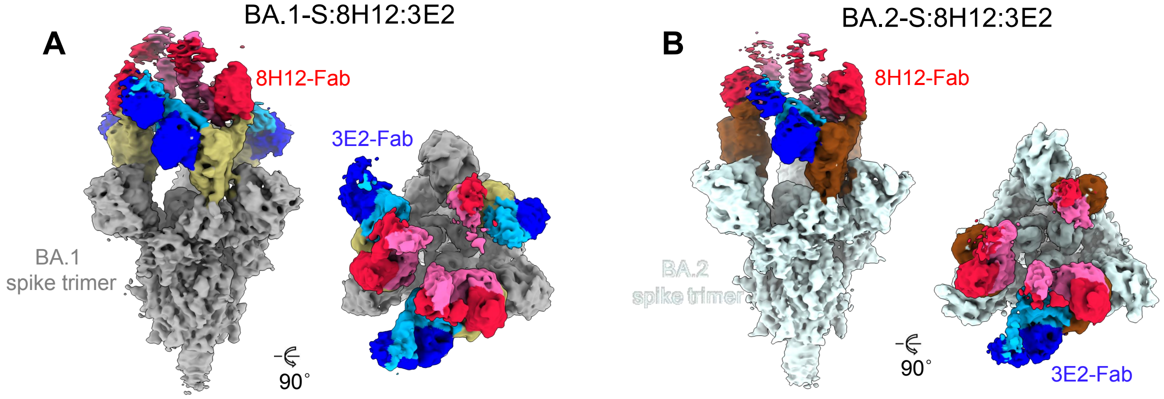
BA.1-S:8H12:3E2
A
8H12-Fab
3E2-Fab
BA.1
spike trimer
90°
BA.2-S:8H12:3E2
B
8H12-Fab
BA.2
spike trimer
90°
3E2-Fab

Fig. S7

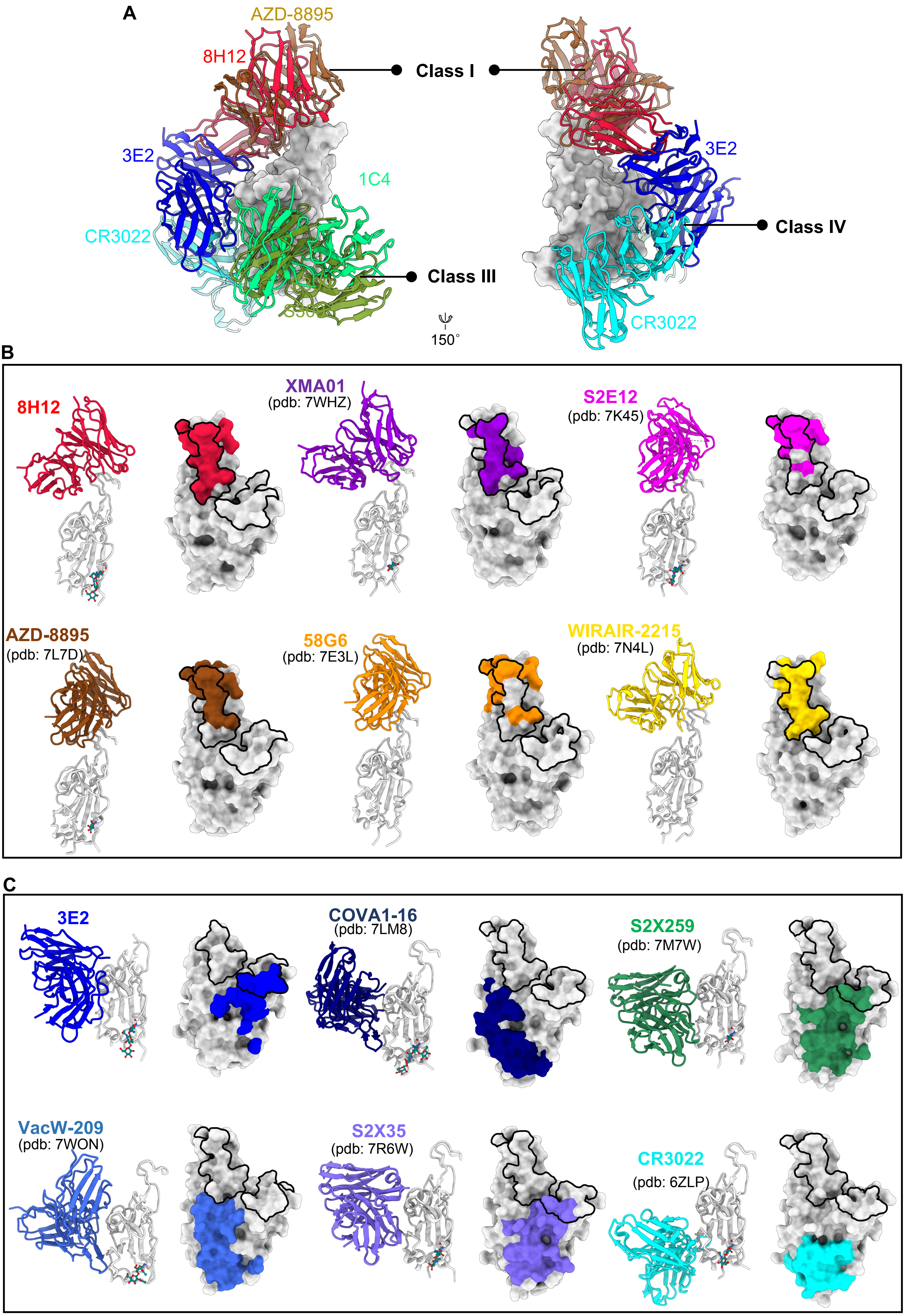
A
AZD-8895
8H12
Class I
3E2
1C4
CR3022
Class III
Class IV
150°
B
8H12
XMA01
(pdb: 7WHZ)
S2E12
(pdb: 7K45)
AZD-8895
(pdb: 7L7D)
58G6
(pdb: 7E3L)
WIRAIR-2215
(pdb: 7N4L)
C
3E2
COVA1-16
(pdb: 7LM8)
S2X259
(pdb: 7M7W)
VacW-209
(pdb: 7WON)
S2X35
(pdb: 7R6W)
CR3022
(pdb: 6ZLP)

Fig. S8

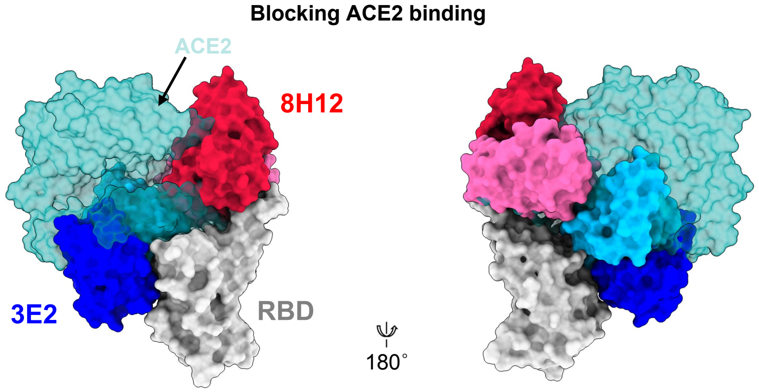

Fig. S9

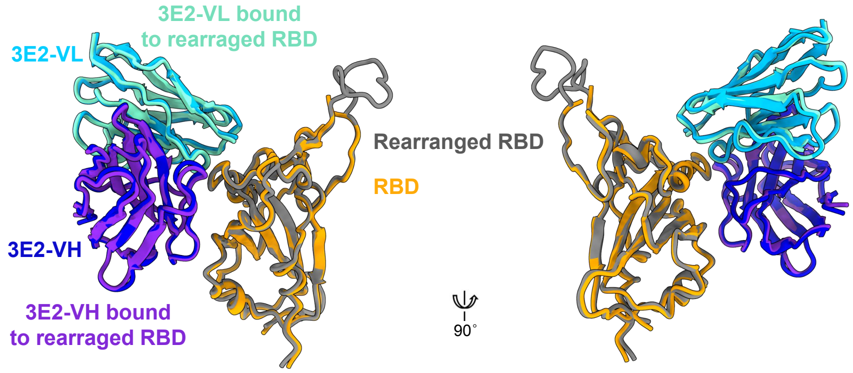
3E2-VL bound
to rearraged RBD
3E2-VL
Rearranged RBD
RBD
3E2-VH
3E2-VH bound
to rearraged RBD
90°

Fig. S10

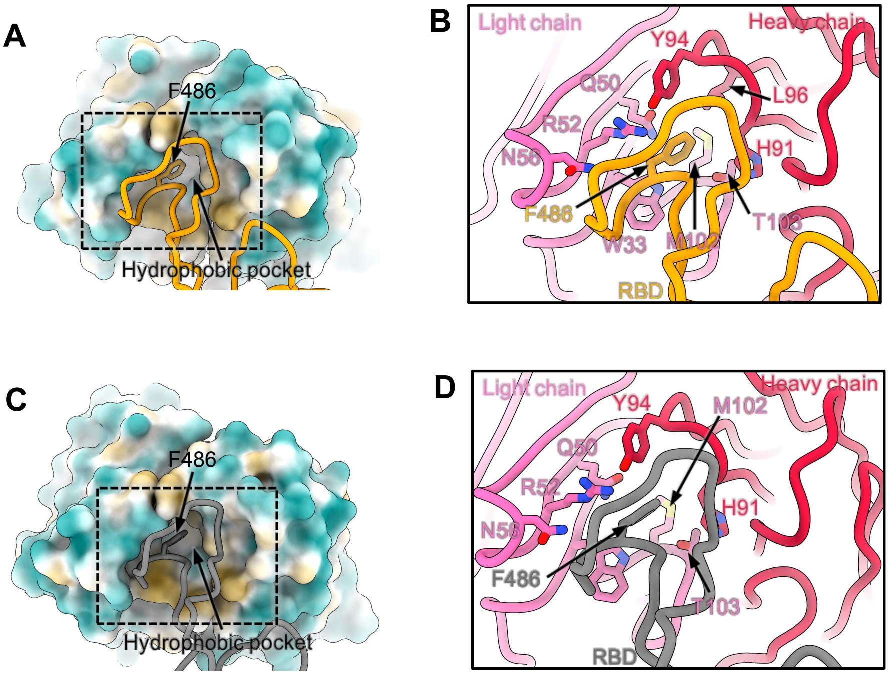
A
F486
Hydrophobic pocket
B
Light chain
Heavy chain
Y94
Q50
L96
R52
H91
N56
F486
W33
M102
T103
RBD
C
F486
Hydrophobic pocket
D
Light chain
Heavy chain
Y94
M102
Q50
R52
H91
N56
F486
T103
RBD

Fig. S11

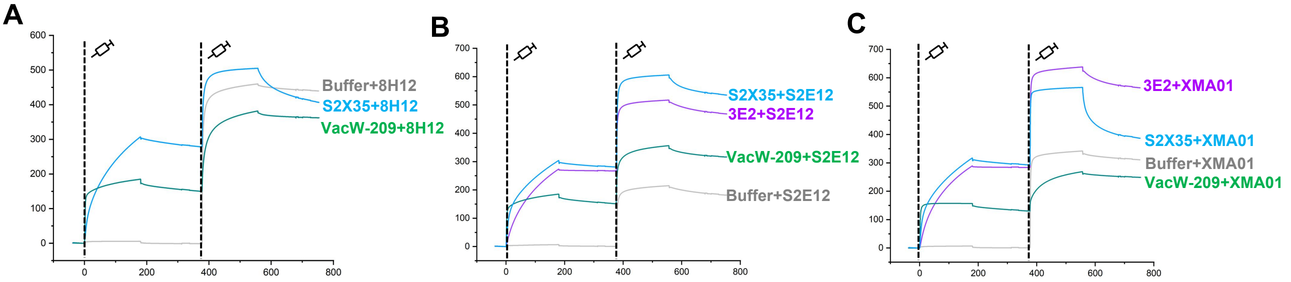
A
B
C
Buffer+8H12
S2X35+8H12
VacW-209+8H12
S2X35+S2E12
3E2+S2E12
VacW-209+S2E12
Buffer+S2E12
3E2+XMA01
S2X35+XMA01
Buffer+XMA01
VacW-209+XMA01
600
500
400
300
200
100
0
700
0
200
400
600
800

Fig. S12

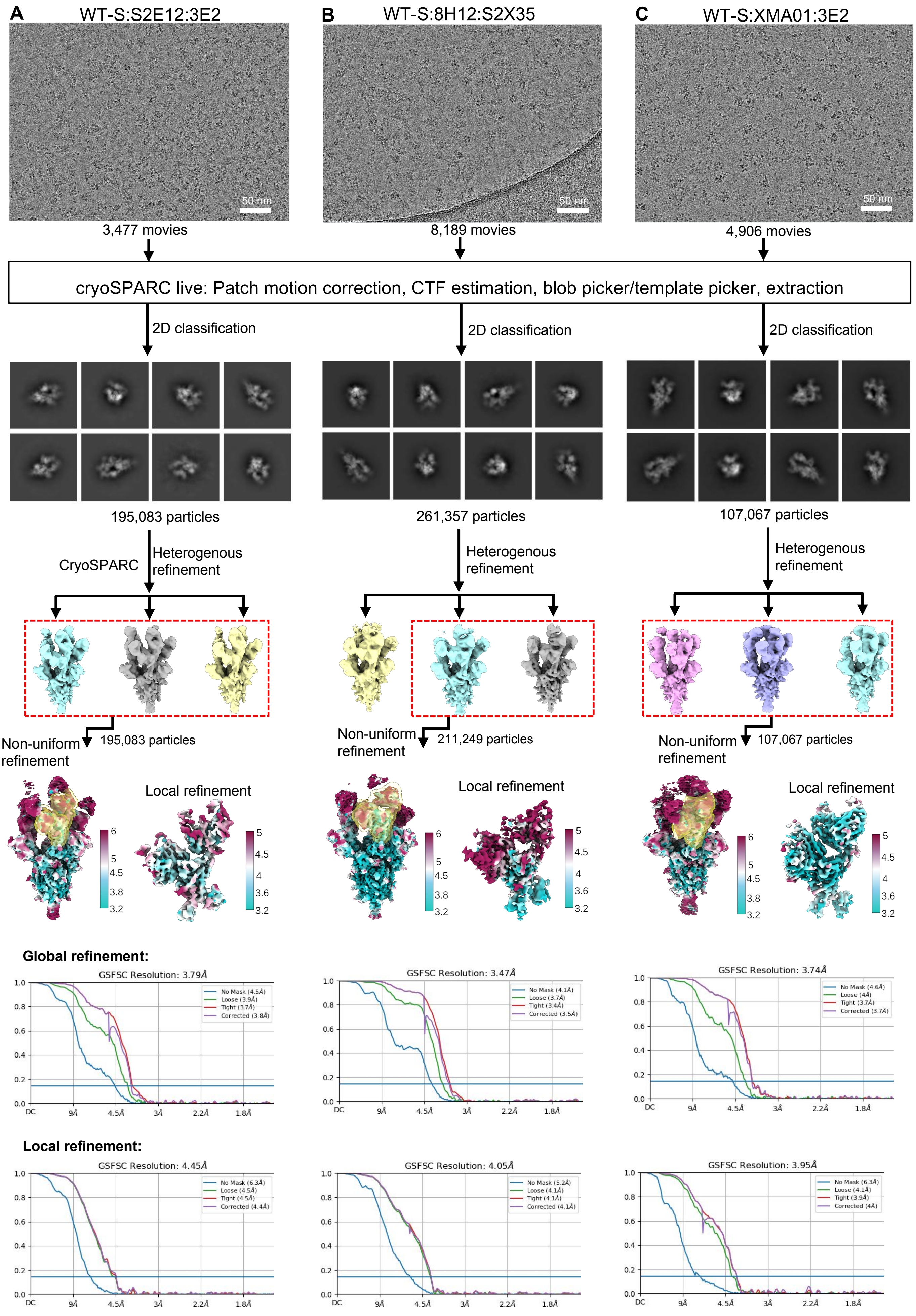

A WT-S:S2E12:3E2
B WT-S:8H12:S2X35
C WT-S:XMA01:3E2
50 nm
3,477 movies
8,189 movies
4,906 movies
cryoSPARC live: Patch motion correction, CTF estimation, blob picker/template picker, extraction
2D classification
195,083 particles
261,357 particles
107,067 particles
CryoSPARC
Heterogenous refinement
Non-uniform refinement
195,083 particles
211,249 particles
107,067 particles
Local refinement
Global refinement:
GSFSC Resolution: 3.79Å
GSFSC Resolution: 3.47Å
GSFSC Resolution: 3.74Å
Local refinement:
GSFSC Resolution: 4.45Å
GSFSC Resolution: 4.05Å
GSFSC Resolution: 3.95Å
No Mask
Loose
Tight
Corrected

Fig. S13

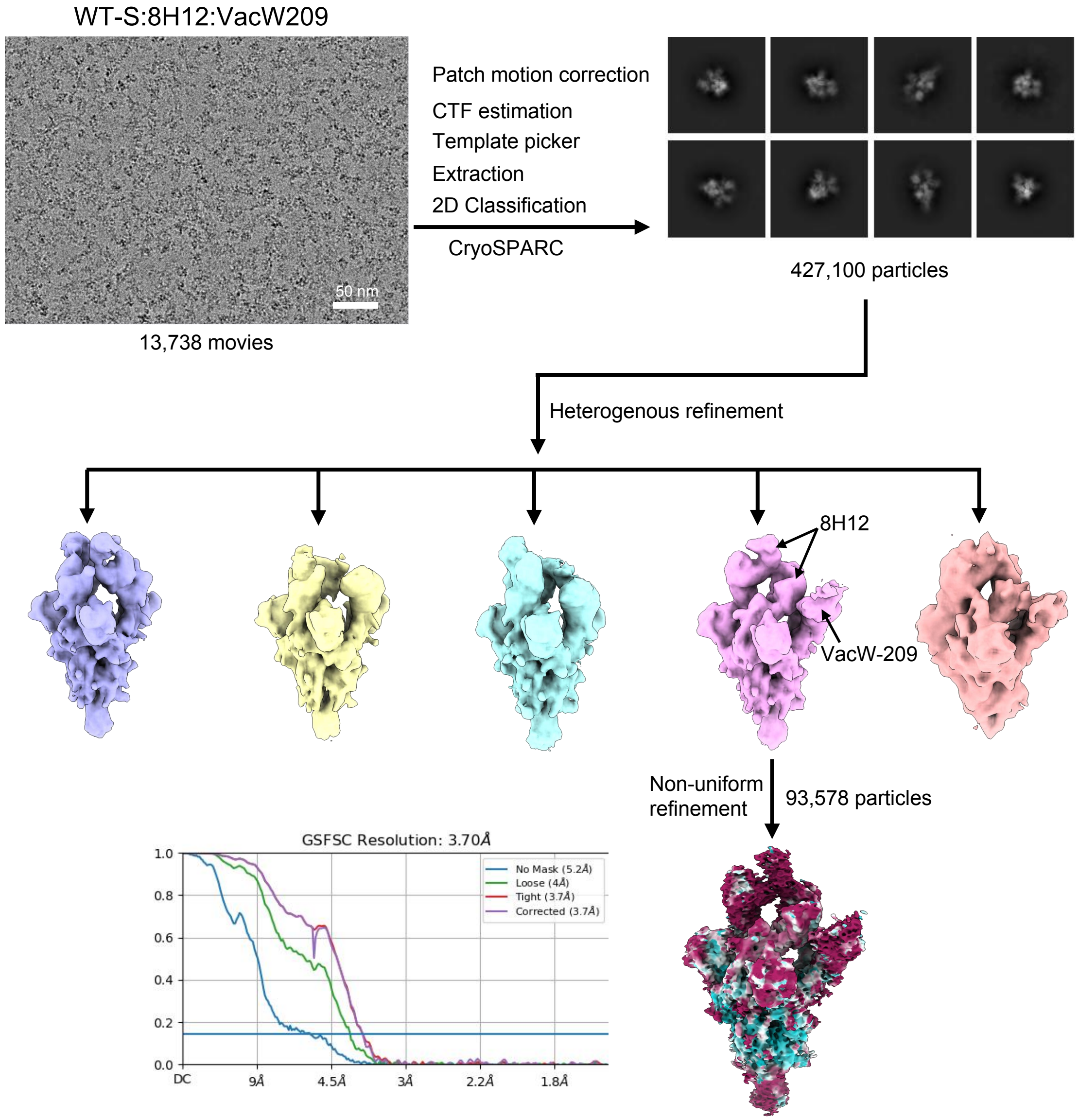
WT-S:8H12:VacW209
50 nm
13,738 movies
Patch motion correction
CTF estimation
Template picker
Extraction
2D Classification
CryoSPARC
427,100 particles
Heterogenous refinement
8H12
VacW-209
Non-uniform refinement
93,578 particles
GSFSC Resolution: 3.70Å
No Mask (5.2Å)
Loose (4Å)
Tight (3.7Å)
Corrected (3.7Å)
1.0
0.8
0.6
0.4
0.2
0.0
DC
9Å
4.5Å
3Å
2.2Å
1.8Å

Fig. S14

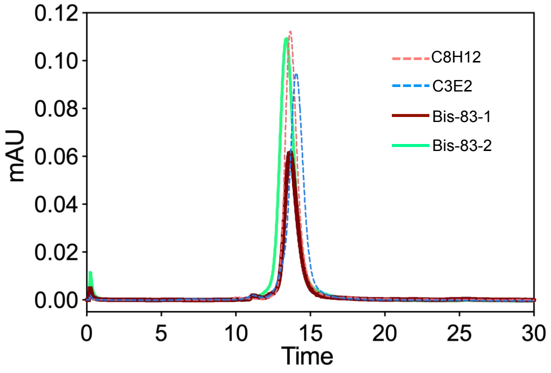

0.12
0.10
0.08
0.06
0.04
0.02
0.00
mAU
0
5
10
15
20
25
30
Time
C8H12
C3E2
Bis-83-1
Bis-83-2

Fig. S15

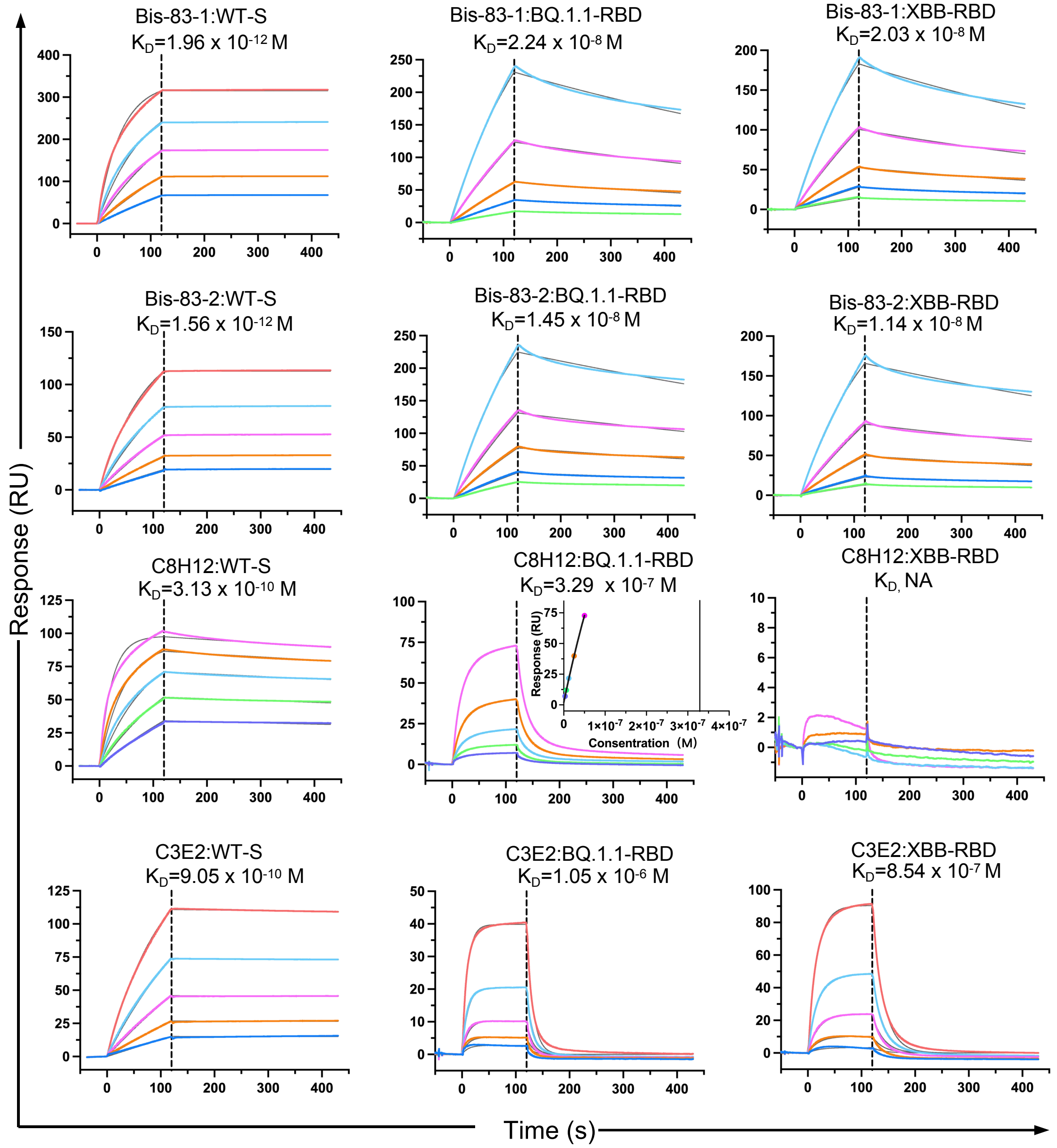
Bis-83-1:WT-S
K_D=1.96 x 10^{-12} M
Bis-83-1:BQ.1.1-RBD
K_D=2.24 x 10^{-8} M
Bis-83-1:XBB-RBD
K_D=2.03 x 10^{-8} M
Bis-83-2:WT-S
K_D=1.56 x 10^{-12} M
Bis-83-2:BQ.1.1-RBD
K_D=1.45 x 10^{-8} M
Bis-83-2:XBB-RBD
K_D=1.14 x 10^{-8} M
C8H12:WT-S
K_D=3.13 x 10^{-10} M
C8H12:BQ.1.1-RBD
K_D=3.29 x 10^{-7} M
Response (RU)
1×10⁻⁷ 2×10⁻⁷ 3×10⁻⁷ 4×10⁻⁷
Consentration (M)
C8H12:XBB-RBD
K_D, NA
C3E2:WT-S
K_D=9.05 x 10^{-10} M
C3E2:BQ.1.1-RBD
K_D=1.05 x 10^{-6} M
C3E2:XBB-RBD
K_D=8.54 x 10^{-7} M
Response (RU)
Time (s)

Fig. S16

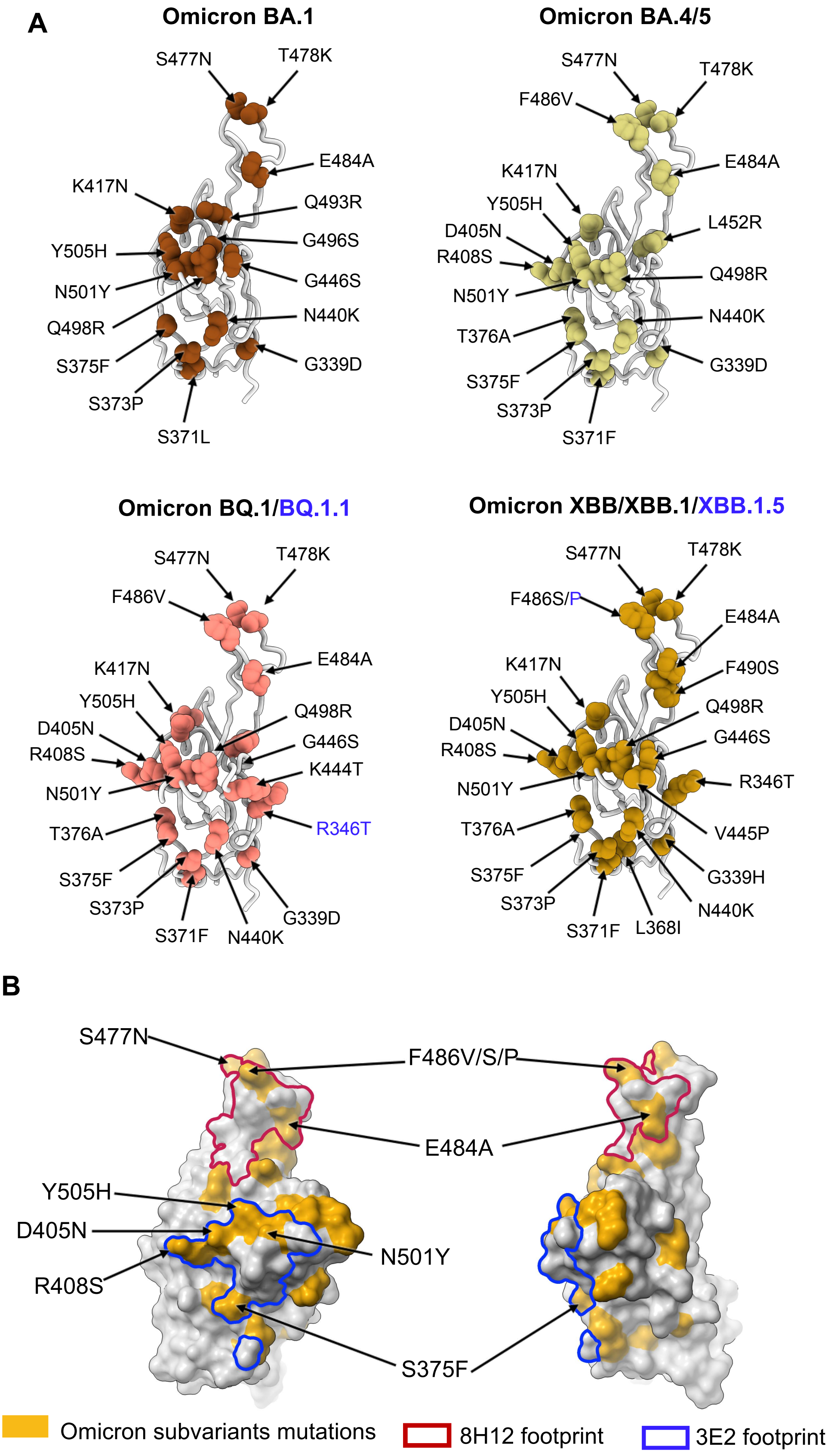
A
Omicron BA.1
S477N
T478K
E484A
K417N
Q493R
G496S
Y505H
G446S
N501Y
N440K
Q498R
G339D
S375F
S373P
S371L
Omicron BA.4/5
S477N
T478K
F486V
E484A
K417N
Y505H
L452R
D405N
R408S
Q498R
N501Y
N440K
T376A
G339D
S375F
S373P
S371F
Omicron BQ.1/BQ.1.1
S477N
T478K
F486V
E484A
K417N
Y505H
Q498R
D405N
G446S
R408S
K444T
N501Y
R346T
T376A
S375F
S373P
G339D
S371F
N440K
Omicron XBB/XBB.1/XBB.1.5
S477N
T478K
F486S/P
E484A
K417N
F490S
Y505H
Q498R
D405N
G446S
R408S
R346T
N501Y
T376A
V445P
S375F
G339H
S373P
N440K
S371F
L368I
B
S477N
F486V/S/P
E484A
Y505H
D405N
N501Y
R408S
S375F
Omicron subvariants mutations
8H12 footprint
3E2 footprint

SARS-CoV-2 strains	IC$_{50}$ (ng/mL)			
	**8H12**	**3E2**	**8H12+3E2**	**S309**
**D614G**	34	160	9	57
**B.1.1.7**	41	151	8	165
**B.1.351**	78	152	21	39
**P.1**	92	140	6	11
**B.1.617.2**	51	219	17	48
**B.1.429**	30	185	6	22
**B.1.525**	100	231	18	17
**B.1.526a**	44	153	13	49
**B.1.526b**	113	263	40	43
**B.1.617.1**	83	347	22	71
**A.VOI.V2**	137	355	44	25
**C.37**	27	145	15	28
**B.1.529**	163	280	119	214
**BA.2**	175	>45,000	244	127
**BA.2.12.1**	34	>45,000	509	1,170
**BA.2.75**	18	>45,000	35	275
**BA.4**	>45,000	>45,000	>45,000	1,023
**BA.5**	>45,000	>45,000	>45,000	636

IC$_{50}$ (ng/mL)

<10	<100	<1,000	<10,000	>45,000
