# Supplementary material for: Two antibodies show broad, synergistic neutralization against SARS-CoV-2 variants by inducing conformational change within the RBD
Source: Protein Cell. 2023 Jul 20;15(2):121–34. doi: 10.1093/procel/pwad040 (PMC10833452; doi:10.1093/procel/pwad040)
Supplement: pwad040_suppl_Supplementary_Tabel_S2 [file pwad040_suppl_supplementary_tabel_s2.pptx]

## Slide 1
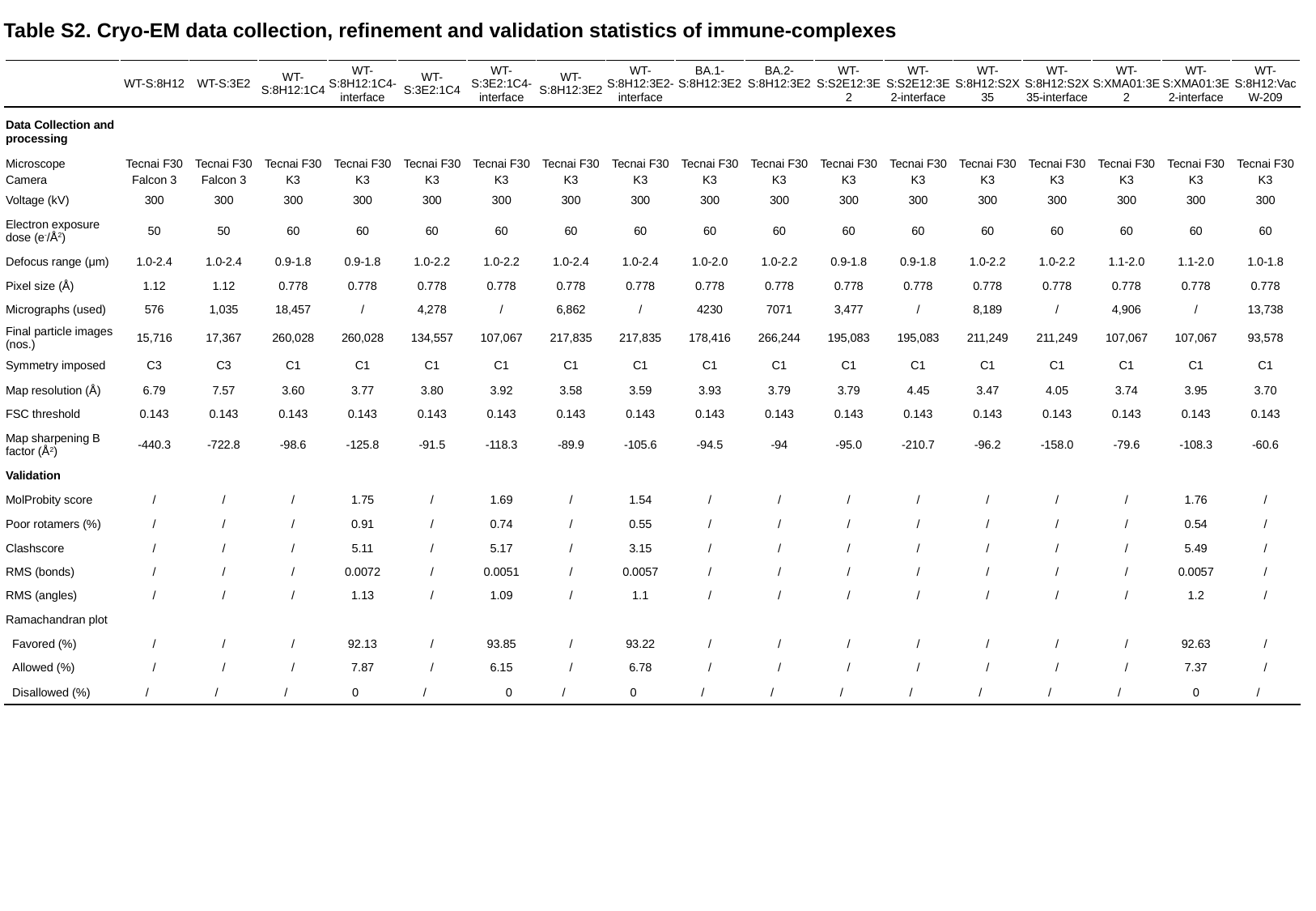

Table S2. Cryo-EM data collection, refinement and validation statistics of immune-complexes
| | WT-S:8H12 | WT-S:3E2 | WT-S:8H12:1C4 | WT-S:8H12:1C4-interface | WT-S:3E2:1C4 | WT-S:3E2:1C4-interface | WT-S:8H12:3E2 | WT-S:8H12:3E2-interface | BA.1-S:8H12:3E2 | BA.2-S:8H12:3E2 | WT-S:S2E12:3E2 | WT-S:S2E12:3E2-interface | WT-S:8H12:S2X35 | WT-S:8H12:S2X35-interface | WT-S:XMA01:3E2 | WT-S:XMA01:3E2-interface | WT-S:8H12:VacW-209 |
| --- | --- | --- | --- | --- | --- | --- | --- | --- | --- | --- | --- | --- | --- | --- | --- | --- | --- |
| Data Collection and processing | | | | | | | | | | | | | | | | | |
| Microscope | Tecnai F30 | Tecnai F30 | Tecnai F30 | Tecnai F30 | Tecnai F30 | Tecnai F30 | Tecnai F30 | Tecnai F30 | Tecnai F30 | Tecnai F30 | Tecnai F30 | Tecnai F30 | Tecnai F30 | Tecnai F30 | Tecnai F30 | Tecnai F30 | Tecnai F30 |
| Camera | Falcon 3 | Falcon 3 | K3 | K3 | K3 | K3 | K3 | K3 | K3 | K3 | K3 | K3 | K3 | K3 | K3 | K3 | K3 |
| Voltage (kV) | 300 | 300 | 300 | 300 | 300 | 300 | 300 | 300 | 300 | 300 | 300 | 300 | 300 | 300 | 300 | 300 | 300 |
| Electron exposure dose (e-/Å2) | 50 | 50 | 60 | 60 | 60 | 60 | 60 | 60 | 60 | 60 | 60 | 60 | 60 | 60 | 60 | 60 | 60 |
| Defocus range (μm) | 1.0-2.4 | 1.0-2.4 | 0.9-1.8 | 0.9-1.8 | 1.0-2.2 | 1.0-2.2 | 1.0-2.4 | 1.0-2.4 | 1.0-2.0 | 1.0-2.2 | 0.9-1.8 | 0.9-1.8 | 1.0-2.2 | 1.0-2.2 | 1.1-2.0 | 1.1-2.0 | 1.0-1.8 |
| Pixel size (Å) | 1.12 | 1.12 | 0.778 | 0.778 | 0.778 | 0.778 | 0.778 | 0.778 | 0.778 | 0.778 | 0.778 | 0.778 | 0.778 | 0.778 | 0.778 | 0.778 | 0.778 |
| Micrographs (used) | 576 | 1,035 | 18,457 | / | 4,278 | / | 6,862 | / | 4230 | 7071 | 3,477 | / | 8,189 | / | 4,906 | / | 13,738 |
| Final particle images (nos.) | 15,716 | 17,367 | 260,028 | 260,028 | 134,557 | 107,067 | 217,835 | 217,835 | 178,416 | 266,244 | 195,083 | 195,083 | 211,249 | 211,249 | 107,067 | 107,067 | 93,578 |
| Symmetry imposed | C3 | C3 | C1 | C1 | C1 | C1 | C1 | C1 | C1 | C1 | C1 | C1 | C1 | C1 | C1 | C1 | C1 |
| Map resolution (Å) | 6.79 | 7.57 | 3.60 | 3.77 | 3.80 | 3.92 | 3.58 | 3.59 | 3.93 | 3.79 | 3.79 | 4.45 | 3.47 | 4.05 | 3.74 | 3.95 | 3.70 |
| FSC threshold | 0.143 | 0.143 | 0.143 | 0.143 | 0.143 | 0.143 | 0.143 | 0.143 | 0.143 | 0.143 | 0.143 | 0.143 | 0.143 | 0.143 | 0.143 | 0.143 | 0.143 |
| Map sharpening B factor (Å2) | -440.3 | -722.8 | -98.6 | -125.8 | -91.5 | -118.3 | -89.9 | -105.6 | -94.5 | -94 | -95.0 | -210.7 | -96.2 | -158.0 | -79.6 | -108.3 | -60.6 |
| Validation | | | | | | | | | | | | | | | | | |
| MolProbity score | / | / | / | 1.75 | / | 1.69 | / | 1.54 | / | / | / | / | / | / | / | 1.76 | / |
| Poor rotamers (%) | / | / | / | 0.91 | / | 0.74 | / | 0.55 | / | / | / | / | / | / | / | 0.54 | / |
| Clashscore | / | / | / | 5.11 | / | 5.17 | / | 3.15 | / | / | / | / | / | / | / | 5.49 | / |
| RMS (bonds) | / | / | / | 0.0072 | / | 0.0051 | / | 0.0057 | / | / | / | / | / | / | / | 0.0057 | / |
| RMS (angles) | / | / | / | 1.13 | / | 1.09 | / | 1.1 | / | / | / | / | / | / | / | 1.2 | / |
| Ramachandran plot | | | | | | | | | | | | | | | | | |
| Favored (%) | / | / | / | 92.13 | / | 93.85 | / | 93.22 | / | / | / | / | / | / | / | 92.63 | / |
| Allowed (%) | / | / | / | 7.87 | / | 6.15 | / | 6.78 | / | / | / | / | / | / | / | 7.37 | / |
| Disallowed (%) | / | / | / | 0 | / | 0 | / | 0 | / | / | / | / | / | / | / | 0 | / |
